# A Highly Porous Nanofibrillar PEDOT:PSS Matrix for Beyond‐Surface Precious‐Metal Utilization and Volumetric Electrocatalysis

**DOI:** 10.1002/smll.202514951

**Published:** 2026-03-31

**Authors:** Da‐Young Lee, Hye‐Min Shin, Ji Hwan Kim, Myung‐Han Yoon

**Affiliations:** ^1^ Department of Materials Science and Engineering Gwangju Institute of Science and Technology (GIST) Gwangju Republic of Korea; ^2^ GIST InnoCORE AI‐Nano Convergence Institute for Early Detection of Neurodegenerative Diseases Gwangju Institute of Science and Technology (GIST) Gwangju Republic of Korea

**Keywords:** ion permeability, Organic mixed ionic‐electronic conductors, PEDOT:PSS, precious metal utilization, volumetric electrocatalysis

## Abstract

Efficient precious‐metal utilization in electrocatalysis requires electrically conductive support architectures that enable electrocatalytic activity beyond conventional surface‐limited designs. Herein, we demonstrate that solvent‐assisted crystallization converts poly(3,4‐ethylenedioxythiophene):poly(styrenesulfonate) (PEDOT:PSS) into a water‐stable, highly conductive nanofibrillar matrix featuring nanoscale porosity and controlled swelling. During the subsequent electrodeposition of Pt nanoparticles (NPs), the highly porous nanofibrillar PEDOT:PSS moderately swells in aqueous media, allowing Pt ions to deeply infiltrate the polymer network and form uniformly dispersed Pt NPs throughout the entire film volume rather than on the surface alone. This volumetric nanoconfinement effect yields a markedly enlarged electrochemically active surface area (20 m^2^ g_Pt_^−1^), rapid reactant permeability, and structural robustness under operating conditions. The resulting PEDOT:PSS–Pt nanocomposite exhibits greatly enhanced catalytic activity for hydrogen evolution and methanol oxidation reactions, outperforming conventional planar Pt architectures. This work establishes highly porous nanofibrillar PEDOT:PSS as a previously underutilized volumetric electrocatalyst scaffold and provides a general design strategy for maximizing precious‐metal efficiency in electrocatalysis and water‐splitting systems.

## Graphical abstract

Solvent‐assisted crystallization transforms PEDOT:PSS into a conductive nanoporous scaffold that enables volumetric incorporation and utilization of Pt nanoparticles. Unlike surface‐limited Pt only electrodes, the PP–Pt electrode allows ion penetration and bulk catalyst activation, improving precious‐metal utilization and sustaining electrocatalytic performance for hydrogen evolution and methanol oxidation.

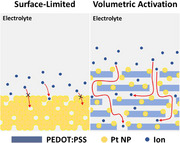

## 1 Introduction

The increasing global energy demand and the environmental consequences associated with the combustion of fossil fuels—including greenhouse gas emissions, air pollution, and the depletion of finite reserves—have accelerated the pursuit of clean, efficient, and sustainable energy technologies [1, 2]. Electrochemical energy‐conversion systems have emerged as a promising solution because they can generate electricity or fuels without combustion by exploiting naturally abundant atmospheric species [3, 4, 5, 6]. Key reactions in these systems, such as hydrogen evolution reaction (HER) [7], oxygen evolution reaction (OER) [8], oxygen reduction reaction (ORR) [9], carbon dioxide reduction reaction (CO_2_RR) [10], and methanol/ethanol oxidation reactions (MOR/EOR) [11, 12], require electrocatalysts that are both highly active and durable. Among the various materials explored, Pt‐based electrocatalysts remain benchmark performers due to their exceptional electrochemical activity and robustness/stability [13, 14]. However, the high cost and scarcity of Pt severely restrict widespread implementation, while many non‐precious metal alternatives suffer from insufficient activity, poor long‐term stability, and incomplete mechanistic understanding [15, 16, 17, 18, 19]. Therefore, maximizing Pt utilization and durability while minimizing overall Pt loading remains a central challenge.

A widely employed strategy to improve Pt utilization is size reduction, which exposes a larger fraction of active surface sites and, thereby, enhances catalytic activity per mass of Pt [20, 21, 22, 23, 24]. Numerous Pt nanostructures have been developed based on this principle, benefiting from high surface area and conductive networks that promote electron transport [25, 26, 27]. Nonetheless, nanoscale Pt typically contributes only through the particles accessible at the external surface of the catalyst layer, leading to limited mass activity despite their small particle size [23, 28]. Further, single‐atom Pt catalysts have been introduced to further maximize atomic efficiency [29, 30, 31], yet their synthetic procedures are costly, low‐yielding, and difficult to scale up. More critically, both single‐atom and nanosized Pt species experience severe aggregation driven by high surface energy, diminishing the number of stable active sites and compromising long‐term catalytic performance [32, 33]. These limitations underscore the need for catalyst designs in which Pt is not merely downsized, but effectively stabilized and uniformly dispersed within a structurally supportive matrix that prevents aggregation while allowing efficient electron transport [33, 34, 35, 36].

More importantly, to truly enhance Pt mass activity, the electrocatalytic reaction must extend beyond the external surface and engage Pt sites distributed throughout a volumetrically larger region of the electrode. Achieving such volumetric electrocatalysis requires support materials that are not only electrically conductive but also structurally suited to enable reactant penetration, maintain mechanical integrity, and host catalytic metal species deep within their frameworks. Various 3D and porous supports spanning diverse material classes—including carbons, metal oxides, and hydrogels—have been investigated to enhance catalyst utilization by increasing the electrochemically accessible surface area [37, 38, 39, 40, 41]. However, these systems often suffer from intrinsic trade‐offs: carbon‐based architectures are typically hydrophobic, which limits deep ion penetration into the bulk; metal oxides offer structural robustness but often lack sufficient electrical conductivity; and traditional polymeric or hydrogel matrices, while ion‐permeable, generally lack the requisite electrochemical stability and electrical conductivity for sustained operation in acidic environments. Furthermore, despite extensive efforts, there exist only few materials which simultaneously provide the controlled swelling behavior, favorable metal (i.e., active catalytic sites)‐matrix interactions, and high mixed ionic/electronic conductivity required for true volumetric electrocatalysis.

In this research, we report a highly crystallized but porous nanofibrillar poly(3,4‐ethylenedioxythiophene):poly(styrenesulfonate) (PEDOT:PSS) matrix that uniquely fulfills the abovementioned criteria. Solvent‐assisted crystallization using sulfuric acid transforms PEDOT:PSS into a highly porous nanofibrillar scaffold with controlled swelling and decent electrical/ionic conductivity, enabling platinum ions to infiltrate and undergo electrochemical reduction throughout the entire film thickness. This swelling‐mediated volumetric incorporation of Pt ions results in a true 3D PEDOT:PSS–Pt nanocomposite. This structure facilitates enhanced utilization of Pt active sites, distinguishing it from the conventional surface‐limited Pt deposition typically observed in most supported catalysts. The resultant PEDOT:PSS–Pt nanocomposite exhibits a large electrochemically active surface area, high reactant permeability, and structural robustness/stability under aqueous electrochemical reaction conditions. Furthermore, we systematically investigate the structural, optical, electrical, and electrochemical characteristics of this hybrid system, demonstrating the previously unexplored potential of PEDOT:PSS as an effective volumetric electrocatalyst support.

## 2 Results and Discussion

**FIGURE 1:**
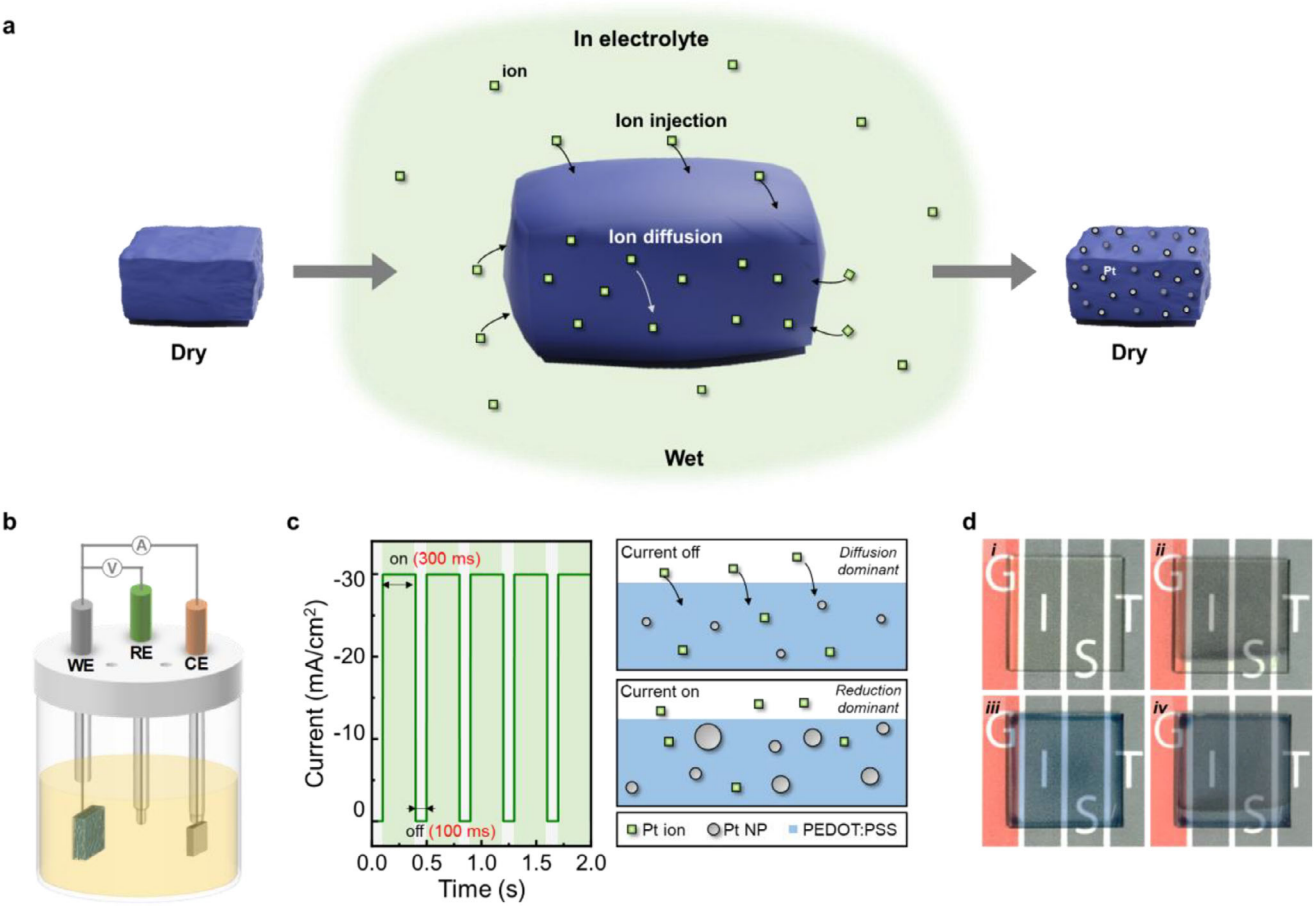
(a) Schematic illustration of fabricating crystalline PEDOT:PSS–Pt nanocomposite film. (b) Schematic illustration of the electrochemical setup for incorporation of Pt NPs into PEDOT:PSS matrix. (c) Pulse‐current electrodeposition profile and corresponding schematics describing Pt nucleation and nanoparticle growth. (d) Photographs of (i) bare ITO, (ii) ITO with Pt NPs, (iii) crystallized PEDOT:PSS, and (iv) crystallized PEDOT:PSS with Pt NPs.

Highly porous nanofibrillar PEDOT:PSS–Pt nanocomposites were fabricated through direct electrodeposition of Pt nanoparticles (NPs) after immersing crystallized PEDOT:PSS films in an aqueous Pt precursor solution, which eliminates the need for pre‐synthesis of Pt NPs (Figure S1). Briefly, pristine PEDOT:PSS films were prepared on indium tin oxide (ITO) or quartz substrates by spin‐coating and, subsequently, treated with sulfuric acid. As described in our previous research, the post‐treatment with H_2_SO_4_ induces PSS removal as well as PEDOT crystallization, resulting in highly porous nanofibrillar structures with significantly enhanced electrical conductivity [42]. It is noteworthy that resultant nanofibrillar PEDOT:PSS films exhibit controlled swelling in water and high electrical conductivity which are critical for incorporation of metal ions (with water molecules) into the whole volume of PEDOT:PSS film and their electrochemical reduction, respectively, leading to a 3‐D reaction zone (Figure 1a). The actual formation of Pt NPs was then carried out by pulse‐current electrodeposition in a standard three‐electrode configuration while the crystallized PEDOT:PSS film was immersed in an aqueous Pt‐ion precursor solution (Figure 1b,c). The high instantaneous current during the pulse on‐time (‐30 mA cm^−2^, *t*_on_ = 300 ms) promotes rapid Pt nucleation, whereas the off‐time (*t*_off_ = 100 ms) facilitates replenishment of Pt ions near the PEDOT:PSS nanofibrillar surface by diffusion [43]. Note that this pulsed current protocol suppresses dendritic Pt growth and enables the formation of uniformly dispersed Pt NPs with small diameters, in contrast to the coarser Pt morphologies obtained via cyclic voltammetry or direct‐current electrodeposition (Figure S2). For comparison, Pt NPs were also electrodeposited onto bare ITO substrates under identical conditions. As shown in Figure 1d, Pt NPs could be successfully deposited on both PEDOT:PSS/ITO and bare ITO.

**FIGURE 2:**
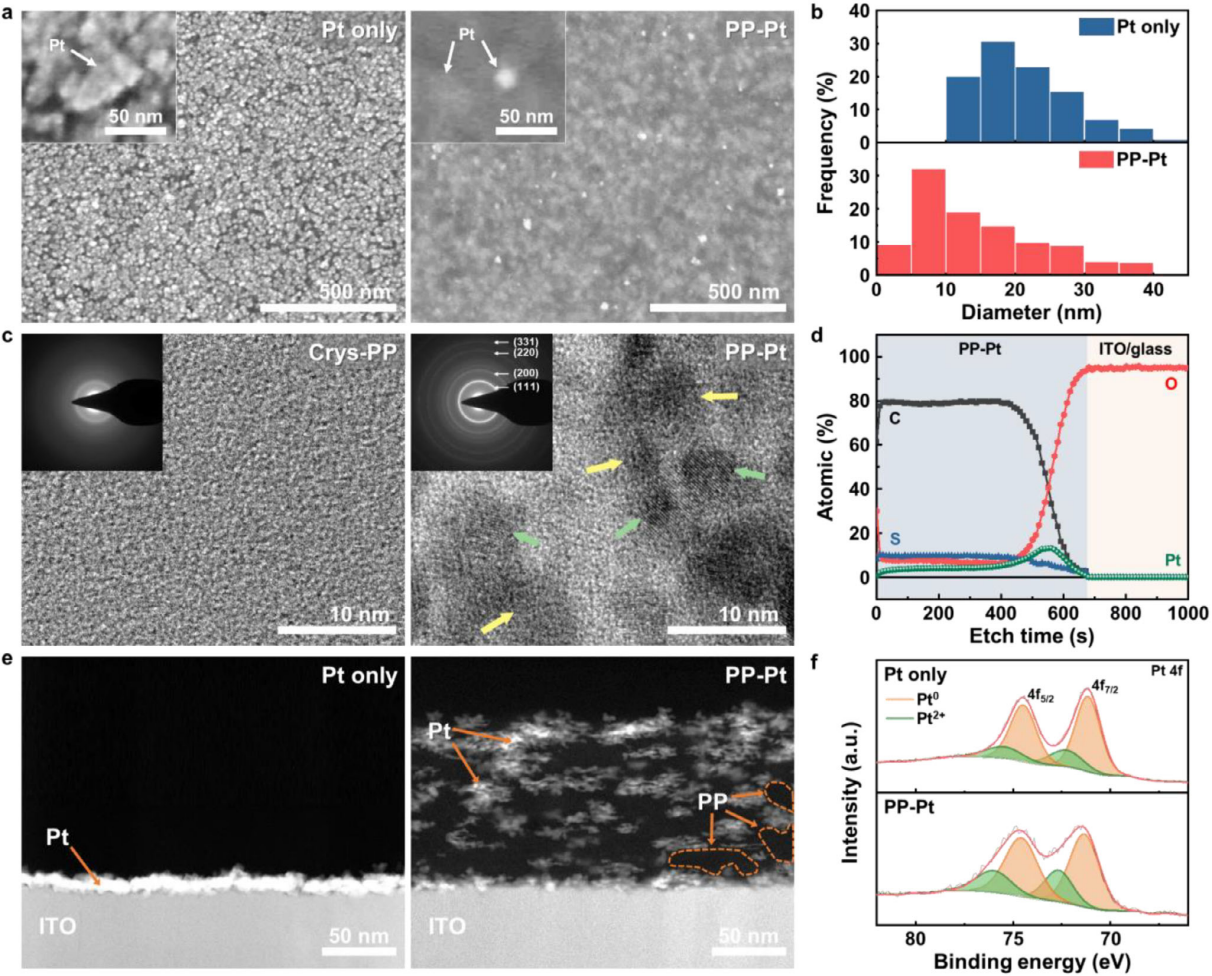
(a) Low‐ and high‐magnification (inset) SEM images of Pt only (left) and PP–Pt (right). (b) Diameter distributions in PP only (upper) and PP–Pt (lower). (c) HRTEM images and corresponding SAED patterns (inset) of crystallized PEDOT:PSS (Crys‐PP; left) and PP–Pt (right). (d) XPS depth profiles of PP–Pt on ITO (C, O, S, Pt elements). (e) Cross‐sectional HAADF‐STEM images of Pt only (left) and PP‐Pt (right). (f) XPS Pt 4*f* peaks of Pt only and PP–Pt (see the main text for more details).

Before evaluating electrocatalytic performance, the structural, optical, and electrical properties of Pt NPs embedded into PEDOT:PSS matrix (PP–Pt) were examined and compared with Pt NPs deposited directly onto bare ITO (Pt only). Scanning electron microscopy (SEM) images (Figure 2a) reveal that both samples exhibit the entire Pt NP coverage; however, PP–Pt displays relatively smaller Pt NPs. Particle‐size analysis (Figure 2b) indicates that the average NP diameters in PP–Pt and Pt only are 14.7 and 21.2 nm, respectively. Notably, the Pt only sample demonstrates more pronounced aggregation, underscoring the critical role of crystallized PEDOT:PSS in suppressing Pt NP aggregation. To examine the distribution of Pt NPs within the composite film, ultra‐high‐resolution SEM in backscattered electron (BSE) mode and high‐angle‐annular dark‐field scanning transmission electron microscopy (HAADF‐STEM) were performed. Both SEM and STEM images of the PP–Pt (Figure S3) consistently reveal high‐contrast signals originating from the Pt NPs within the PP–Pt nanocomposite. These observations suggest that Pt formation is not confined to the film surface but occurs throughout the bulk. Furthermore, HAADF‐STEM coupled with energy‐dispersive X‐ray spectroscopy (EDS) elemental mapping of S and Pt confirms that the Pt signals are uniformly distributed across the composite film, supporting the verification of homogeneous Pt NP incorporation (Figure S4).

High‐resolution transmission electron microscopy (HR‐TEM) confirms that the Pt NPs are highly crystalline, exhibiting lattice spacings corresponding to the (111), (200), (220), and (311) planes. Importantly, Pt NPs are observed both on the surface and in the interior of the crystallized PEDOT:PSS matrix: NPs presumably close to the surface (green arrows in Figure 2c) show distinct Pt(111) *d*‐spacing, whereas those embedded inside (yellow arrows in Figure 2c) exhibit less distinct lattice fringes, possibly due to partial encapsulation within the polymer matrix and limited contrast. Indeed, X‐ray photoelectron spectroscopy (XPS) with depth profiling by argon etching (Figure 2d) further shows the substantial Pt intensities below the surface of PEDOT:PSS film, confirming that Pt NPs exist throughout the bulk film rather than only at skin‐depth the surface. To provide a quantitative interpretation of the depth distribution, the etching time was correlated with the experimentally measured film thickness (∼100 nm). Note that the appearance of the increased O 1*s* signal from the underlying ITO substrate after 670 s of sputtering corresponds to an average etching rate of ∼0.15 nm s^−1^. This calibration enables the conversion of etching time into a physical depth scale. The continuous detection of Pt signals throughout this depth range indicates the volumetric incorporation of Pt NPs within the crystallized PEDOT:PSS matrix. Additionally, cross‐sectional HAADF‐STEM analysis was conducted to directly visualize the spatial distribution of Pt NPs within the architecture of both the PP–Pt and Pt only electrodes (Figure 2e). Consistent with the XPS depth‐profiling results, the PP–Pt electrode exhibits Pt NPs distributed throughout the entire crystallized PEDOT:PSS film confirming volumetric incorporation facilitated by the polymer matrix. In contrast, the Pt only electrode shows a dense Pt layer localized exclusively at the electrode surface, reflecting the surface‐confined growth typical of planar substrates that lack ion‐accessible internal pathways. Cross‐sectional STEM‐EDS elemental mapping (Figure S5) further confirms continuous Pt signals across the full thickness of the PEDOT:PSS in the PP–Pt sample. These observations provide direct structural evidence that the swellable and nanoporous PEDOT:PSS framework enables the penetration of precursor ions and subsequent internal nucleation of Pt NPs. This results in a truly 3D catalytic architecture rather than a conventional surface‐limited catalyst layer. The origin of this volumetric Pt incorporation was investigated by examining the morphology of the crystallized PEDOT:PSS matrix only. BSE‐SEM and HAADF‐STEM images of the crystallized PEDOT:PSS film (Figure S6) reveal a highly porous and interconnected nanofibrillar structure formed during solvent‐assisted crystallization. We suppose that this nanoporous morphology provides ion‐accessible pathways throughout the film, facilitating the penetration of Pt precursor ions during electrodeposition and enabling uniform nucleation within the polymer interior.

The electronic structures of Pt atoms were analyzed by investigating Pt 4*f* peaks in XPS data (Figure 2f). Both PP–Pt and Pt only samples exhibit the characteristic 4*f*_7/2_ and 4*f*_5/2_ doublets, which can be deconvoluted into Pt^0^ and Pt^2+^ species. A positive binding‐energy shift in PP–Pt (71.4 and 74.6 eV) from Pt only (71.1 and 74.4 eV) indicates an electron deficiency in the Pt NPs [44, 45], suggesting strong electronic interactions between Pt and the crystallized PEDOT:PSS matrix that may contribute to the enhanced stabilization of Pt NPs. The electrical conductivity—an important parameter for charge transport during electrocatalysis—was measured using a four‐point probe. Crystallized PEDOT:PSS exhibits substantially enhanced electrical conductivity of ∼2000 S cm^−1^ compared to pristine PEDOT:PSS (∼1 S cm^−^^1^) which is attributed to the removal of excess insulating PSS chains and improved ordering of PEDOT chains induced by sulfuric‐acid treatment (Figure S7) [42, 46]. Note that this conductivity far exceeds that of typical electrocatalyst supports such as carbon (∼30 S cm^−1^) [47, 48] and WO_3‐x_ (1.8 S cm^−1^) [49]. Furthermore, the PP–Pt shows an additional increase in electrical conductivity (∼2200 S cm^−1^) due to the incorporation of conductive Pt NPs within the polymer network.

**FIGURE 3:**
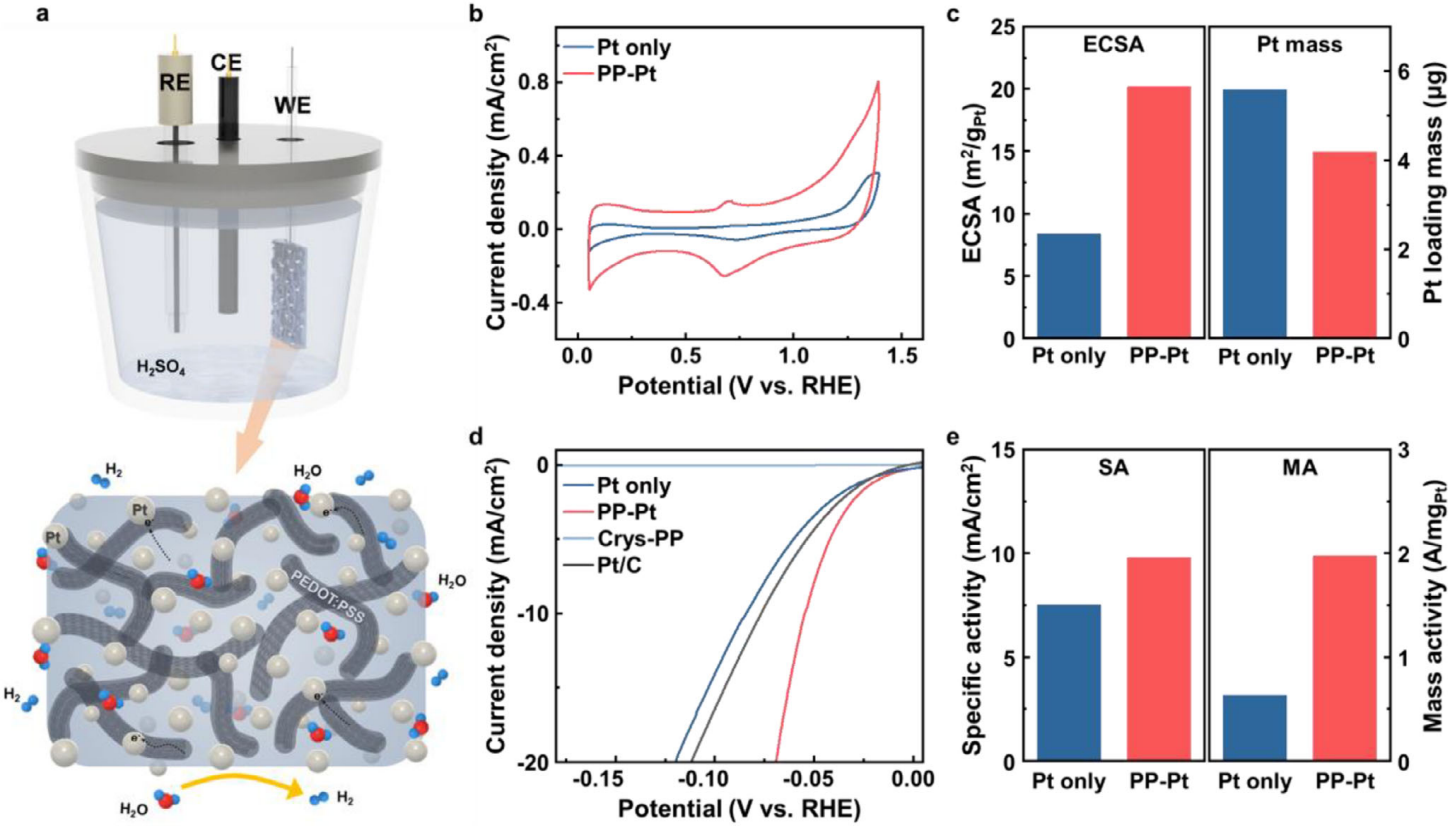
(a) Schematic illustration of the 3‐electrode electrochemical system for HER (upper) and highly porous nanofibrillar PP–Pt nanocomposite. (b) CV curves of Pt only (blue) and PP–Pt (red) (0.5 M H_2_SO_4_, N_2_ purged, scan rate of 50 mV s^−1^). (c) Histograms of ECSA values (left) and Pt loading mass of Pt only and PP–Pt. (d) iR‐corrected LSV curves of Pt only, PP–Pt, crystallized PEDOT:PSS (Crys‐PP), and Pt/C (0.5 M H_2_SO_4_, N_2_ purged, scan rate of 10 mV s^−1^). (e) Histograms of specific (left) and mass activities (right) of Pt only and PP–Pt at an overpotential of 50 mV.

Hydrogen evolution reaction (HER) activities of the PP–Pt and Pt only electrodes were examined using cyclic voltammetry (CV) and linear sweep voltammetry (LSV) in a standard three‐electrode configuration (Figure 3a). CV measurements were conducted in 0.5 M H_2_SO_4_ to determine the electrochemically active surface area (ECSA). As shown in Figure 3b, both electrodes exhibit characteristic hydrogen adsorption and desorption features, yet PP–Pt displays substantially larger hydrogen desorption peak areas. The Pt mass loading quantified by inductively coupled plasma optical emission spectrometry (ICP‐OES) was 4.2 µg for PP–Pt and 5.6 µg for Pt only. ECSA was calculated from the integrated hydrogen desorption charge assuming 0.210 mC cm^−2^ for a monolayer of hydrogen adsorption on Pt. PP–Pt exhibits an ECSA of 20.2 m^2^ g_Pt_^−1^ which is 2.4 times larger than that of Pt only (Figure 3c). This increase could be attributed to more uniform dispersion and smaller size of Pt NP within the PEDOT:PSS scaffold, which suppresses nanoparticle aggregation and enables volumetric electrocatalysis through a highly porous and interconnected nanofibrillar network. Electrochemical impedance spectroscopy (EIS) was performed to evaluate charge‐transfer kinetics during HER (Figure S8). PP–Pt exhibits a markedly lower charge‐transfer resistance (*R*_ct_ = 2.4 Ω) than Pt only (17.6 Ω) which indicates more efficient interfacial electron transfer. In parallel, HER performance was further examined using LSV in acidic media (Figure 3d). After *iR* correction, PP–Pt requires an overpotential of only 54 mV to reach 10 mA cm^−2^, whereas Pt only requires 84 mV. The corresponding Tafel slopes, derived from the Tafel equation (*η* = *b* log *j* + *a*), are 33 mV dec^−1^ for PP–Pt and 57 mV dec^−1^ for Pt only (Figure S9), confirming accelerated reaction kinetics on PP–Pt. Furthermore, for optimizing precious metal utilization, it is essential to assess not only overpotential but also mass activity and specific activity. Figure 3e represents these metrics at an overpotential of 50 mV. PP–Pt shows a mass activity of 2.0 A mg_Pt_^−1^, which is 3.2 times higher than Pt only (0.63 A mg_Pt_^−1^). The pronounced increase in mass activity indicates significantly improved Pt utilization efficiency within the composite matrix. The specific activity of PP–Pt also shows modest improvement relative to Pt only. The greatly enhanced HER performance of PP–Pt arises from suppressed aggregation and improved dispersion of Pt nanoparticles enabled by the highly porous nanofibrillar PEDOT:PSS framework, which increases accessible active sites and facilitates efficient charge transfer. Furthermore, chronopotentiometry measurements performed at 10 and 20 mA cm^−2^ demonstrate the stable HER operation of the PP–Pt electrode under continuous electrolysis. In contrast, the Pt only electrode exhibits rapid potential deterioration during the prolonged operation (Figure S10). This degradation in the Pt only electrode is possibly originated from bubble‐induced catalyst detachment and increased interfacial resistance during gas evolution. Conversely, embedding Pt NPs within the crystallized PEDOT:PSS matrix suppresses detachment and maintains electrochemical stability. These results indicate that the highly porous PEDOT:PSS framework enables efficient volumetric utilization of Pt during the HER process.

**FIGURE 4:**
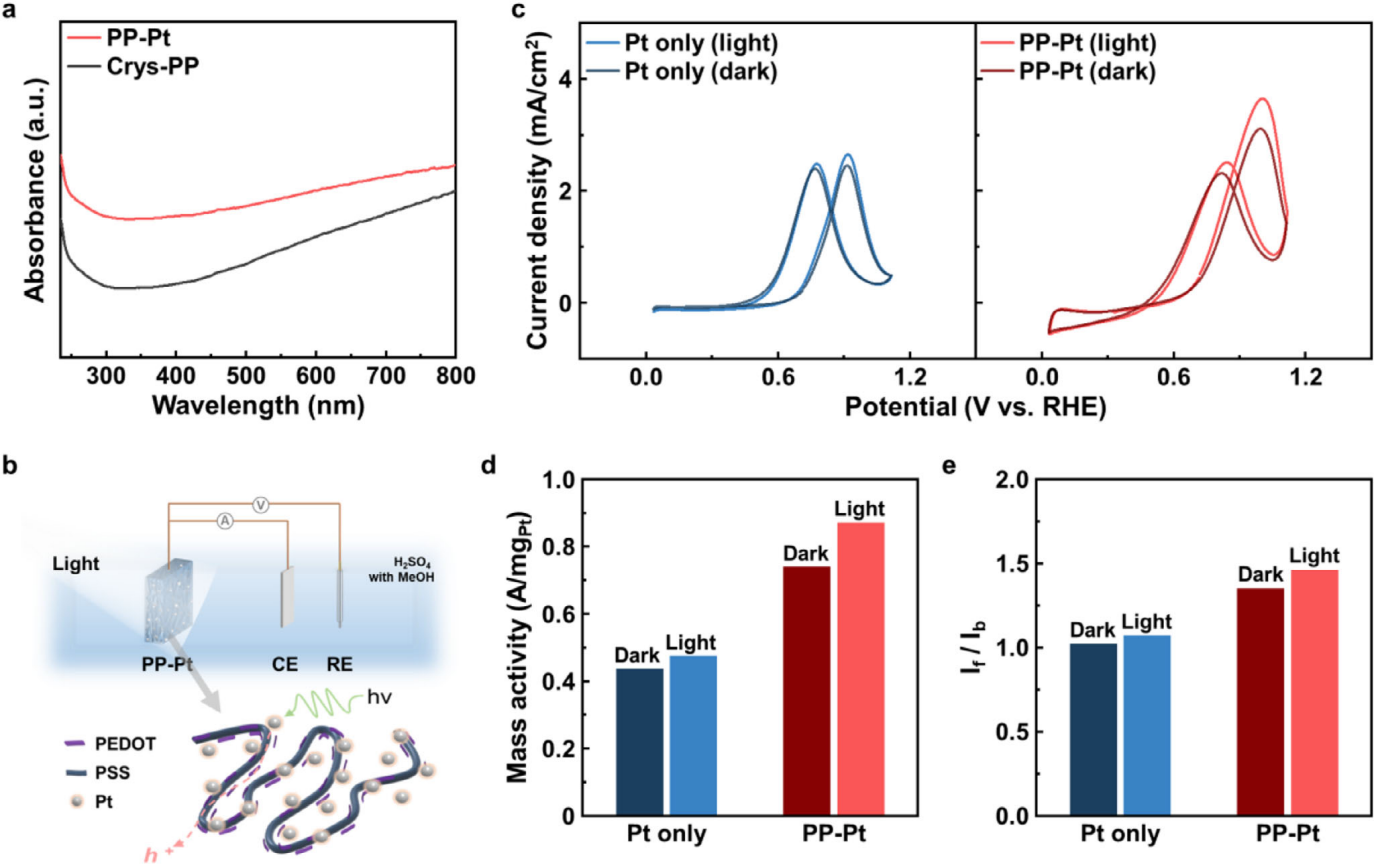
(a) UV–vis absorption spectra of PP–Pt (red) and crystallized PEDOT:PSS films (Crys‐PP; black). (b) Schematic illustration of t the 3‐electrode electrochemical system for methanol oxidation reaction (MOR). (c) CV curves of Pt only (left) and PP–Pt (right) under dark/light condition (0.5 M H_2_SO_4_, N_2_ purged, scan rate of 50 mV s^−1^). (d) Histograms of mass activities of Pt only and PP–Pt under dark/light condition. (e) Histograms of *I*_f_ /*I*_b_ values of Pt only and PP–Pt under dark/light condition.

Next, electrocatalytic activities of PP–Pt and Pt only electrodes toward methanol oxidation, a key reaction for direct methanol fuel cells were examined with/without light irradiation. Note that the PP–Pt electrode exhibits enhanced optical absorbance compared to the crystallized PEDOT:PSS only electrode due to Pt NPs embedded within the polymer matrix (Figure 4a). Despite Pt incorporation, the PP–Pt maintains relatively high optical transmittance (>60%) across the visible range which is almost comparable to that of crystallized PEDOT:PSS only (Figure S11a), indicating that light can effectively penetrate through the electrode thickness. This combination of preserved transparency and increased light absorption suggests efficient light‐matter interaction throughout the composite electrode rather than surface‐limited optical excitation. Raman spectroscopy further reveals changes in the characteristic vibrational profiles of PEDOT following Pt incorporation (Figure S11b). These changes imply a modification of the polymer chain environment and electronic structure, possibly induced by intimate metal‐polymer interactions. Such optical and structural modifications are expected to facilitate the photo‐induced enhancement of electrocatalytic activity under illumination.

Based on these optical characteristics, methanol oxidation reaction (MOR) performance was evaluated in 0.5 M H_2_SO_4_ with 0.1 M CH_3_OH at a scan rate of 50 mV s^−1^ (Figure 4b). The CV curves of PP–Pt and Pt only exhibit two characteristic anodic peaks corresponding to the forward and backward oxidation processes (Figure 4c). The PP–Pt electrode shows a markedly higher forward peak current density than the Pt only electrode, demonstrating superior MOR catalytic activity. Under illumination, the MOR current of PP–Pt further increases, reaching a forward peak current density of 3.65 mA cm^−2^—approximately 1.2 time larger than that in the dark condition. In contrast, the Pt only electrode exhibits only an 8.5% enhancement upon illumination. The enhanced MOR activity under illumination is attributed to photo‐induced effects enabled by the integrated PP–Pt composite structure, possibly involving localized thermal or electronic excitation processes. The mass activities of PP–Pt reach 0.74 A mg_Pt_^−1^ (dark) and 0.87 A mg_Pt_^−1^ (light) (Figure 4d), corresponding to improvements by 1.69 and 1.84 time compared to Pt only, respectively. These results highlight efficient utilization of Pt during the MOR process. It is known that during methanol oxidation, the formation and adsorption of intermediate carbonaceous species poison Pt catalysts [50, 51, 52]. The ratio of the forward anodic peak current (*I*_f_) to the backward anodic peak current (*I*_b_) is widely used as an indicator of poisoning tolerance, with higher *I*_f_/*I*_b_ values reflecting more efficient methanol oxidation and greater resistance to surface poisoning. As shown in Figure 4e, the PP–Pt electrode exhibits the highest *I*_f_/*I*_b_ ratio among the tested catalysts, confirming its superior resistance to carbonaceous‐species accumulation. Chronoamperometry measurements further confirm the stable MOR operation of the PP–Pt electrode under continuous reaction conditions. Following an initial decay period—typically associated with the adsorption of reaction intermediates—the electrode exhibits a well‐defined, stabilized current response (Figure S12).

**FIGURE 5:**
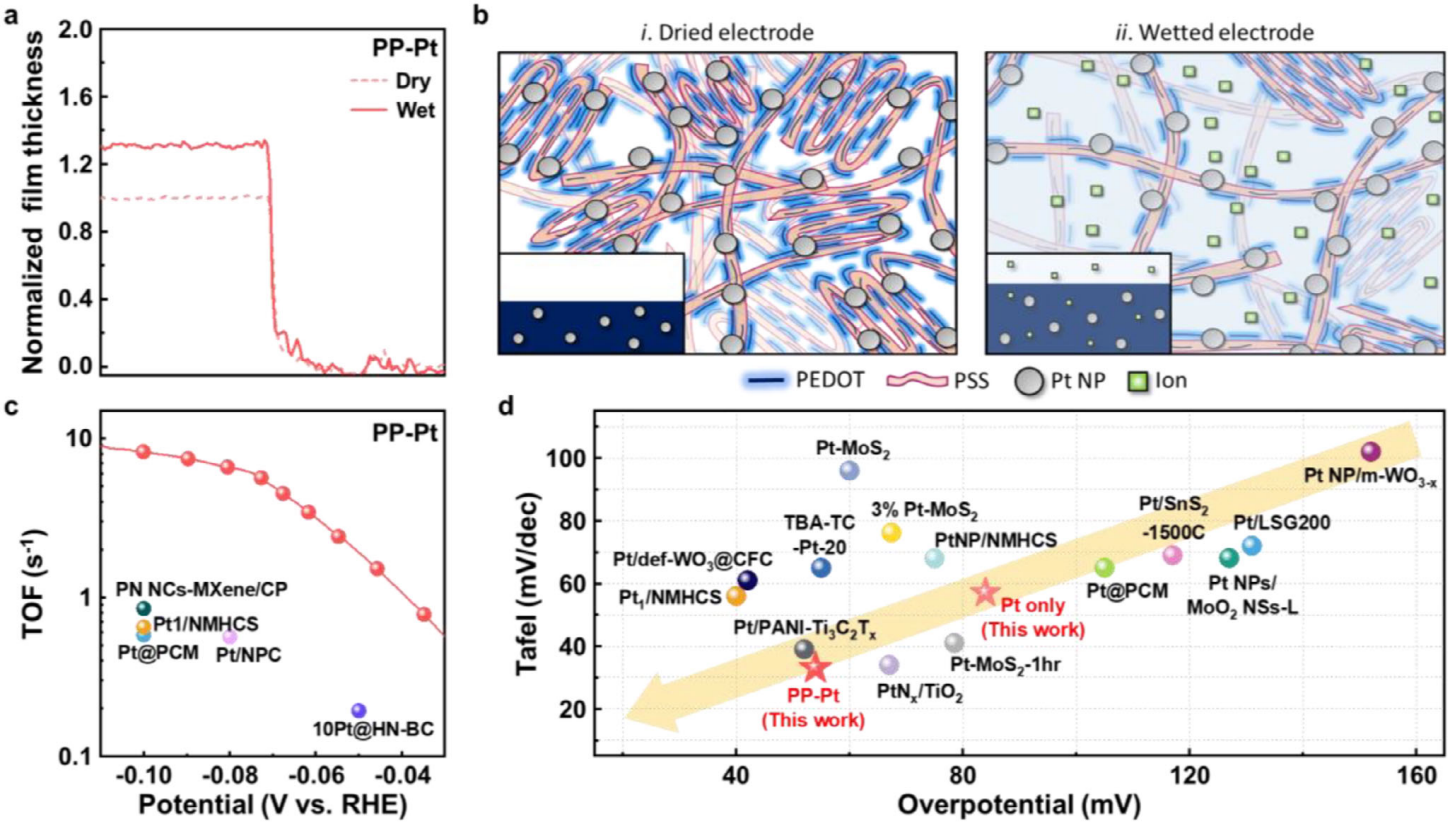
(a) Film thicknesses profiles of PP–Pt films under dry (dotted) and wet conditions (solid) measured by liquid cell AFM. (b) Schematic illustrations of the proposed swelling mechanisms in Pt NP incorporated PEDOT:PSS film under dry (left) and wet (right) conditions. Insets show cross‐sectional view. (c) Plots of turnover frequency (TOF) of PP–Pt and Pt‐based electrocatalysts in the previous literature. (d) Bench marking plots of Tafel slopes vs. overpotential for HER (see the main text for more details).

As depicted in Figure 1, the post‐treatment with sulfuric acid induces solvent‐assisted crystallization in PEDOT:PSS and removes excess insulating PSS chains, resulting in improved robustness/stability in aqueous environments by suppressing uncontrolled swelling [42, 53]. To evaluate the swelling behavior of the films, the thicknesses of dry and wet/swollen PP–Pt, Pt only, crystallized PEDOT:PSS, and pristine PEDOT:PSS were measured using liquid‐cell atomic force microscopy (LC‐AFM) (Figure 5a; Figure S13). Upon immersion in water, the thickness of PP–Pt increased by approximately 30%, while crystallized PEDOT:PSS expanded by 35%. In contrast, Pt only maintained its original thickness but pristine PEDOT:PSS (without sulfuric acid treatment) exhibited dramatic swelling (>1000%) and poor stability, confirming the critical role of solvent‐assisted crystallization in terms of stabilizing the polymer film in aqueous environments.

Now, we propose the mechanism on PP–Pt nanocomposite formation and its enhanced HER/MOR performance based on all experimental evidences (Figure 5b; Figure S14). Upon exposure to aqueous electrolytes, the crystallized PEDOT:PSS film undergoes moderate/controlled swelling that creates pathways for water and metal ion diffusion throughout the entire film volume. When immersed in the Pt precursor solution, Pt ions infiltrate the swollen polymer network and are electrochemically reduced to form Pt NPs which are stably embedded within the matrix (Figure 2e,f). During electrocatalysis in acidic electrolyte, reactant molecules/ions similarly permeate the highly porous nanofibrillar PEDOT:PSS network, allowing catalytic reactions to occur not only on surface‐exposed Pt NPs but also on those located inside the film. This results in a significantly expanded 3‐D reaction volume and a substantially larger effective catalytic area.

In contrast, Pt NPs deposited directly on ITO form dense surface layers lacking internal nanoporosity and swellable domains. Because metallic Pt does not expand or permit ion penetration, reactant molecules/ions and supporting electrolytes can access only the outermost nanoparticles (Figure S14). In fact, although the Pt only sample contains a greater total Pt mass than PP–Pt (Figure 3c), many nanoparticles are buried and electrochemically inaccessible, leading to the inferior HER and MOR performance as shown in Figures 3 and 4. To further compare catalytic efficiency, turnover frequency (TOF)—a loading‐independent figure of merit—was calculated from the polarization curves. At −100 mV vs RHE, PP–Pt exhibits a TOF of 8.25 s^−1^ (Figure 5c; Figure S15; Table S1), substantially higher than Pt (2.55 s^−^^1^) and matching or surpassing the performance of most of previously reported Pt‐based electrocatalysts. Comparison with literature (Figure 5d; Table S2) shows that PP–Pt is comparable or superior to state‐of‐the‐art Pt electrocatalytic systems despite using significantly less amount of Pt. Notably, only a limited number of studies have explored PEDOT:PSS as a catalysis support, and the present research highlights its exceptional capability to enable volumetric electrocatalysis. Overall, the highly porous nanofibrillar PEDOT:PSS scaffold facilitates ion/molecule penetration deep into the electrode, enabling reactions on both surface‐ and bulk embedded‐Pt NPs; this volumetric electrocatalytic behavior dramatically increases the Pt utilization efficiency and underpins the markedly improved HER and MOR activities of PP–Pt.

## 3 Conclusions

In this research, we demonstrated that highly porous nanofibrillar PEDOT:PSS functions as a highly effective volumetric electrocatalyst support that fundamentally enhances the utilization and electrocatalytic activity of Pt nanoparticles. Solvent‐assisted crystallization transforms PEDOT:PSS into a nanofibrillar, porous, and electrically conductive network with excellent aqueous stability and controlled swelling behavior. These characteristics allow deep ion/molecule penetration and uniform nanoparticle nucleation throughout the entire polymer volume, yielding a true 3‐D electrocatalyst architecture rather than a conventional surface‐confined deposition. The resulting crystallized PP–Pt nanocomposite exhibits outstanding electrochemical performance. For HER, it delivers an overpotential of 54.7 mV at 10 mA cm^−2^, an ECSA of 20.2 m^2^ g_Pt_^−1^, and a mass activity of 2.0 A mg_Pt_^−1^—representing 2.4‐fold and 3.2‐fold enhancements, respectively, compared to the Pt only electrode. The resultant nanocomposite also demonstrates markedly improved methanol oxidation activity and a pronounced photocatalytic enhancement under illumination. Importantly, these improvements arise not solely from the presence of Pt, but from the synergistic interplay between Pt NPs and the highly porous nanofibrillar PEDOT:PSS matrix, which provides continuous electronic pathways, large nanoscale porosity, and volumetrically accessible reaction spaces. Considered all together, our research establishes crystallized PEDOT:PSS as a powerful design platform for maximizing precious‐metal efficiency through volumetric electrocatalysis enabled by controlled swelling and high electrical conductivity. The mechanistic principle demonstrated here—embedding metal species within a swellable, conductive polymer scaffold to activate catalytic sites throughout the bulk rather than solely at the surface—provides a materials design strategy that may be extendable to other metal or hybrid electrocatalyst systems beyond Pt. We anticipate that such swelling‐regulated volumetric electrocatalyst architectures with decently high electrical conductivity will enable new opportunities in water splitting, fuel cells, CO_2_ conversion, and other electrochemical technologies where efficient use of scarce catalytic materials is very critical.

## 4 Experimental Section

### 4.1 Preparation of Crystallized PEDOT:PSS–Pt NPs Composite Catalyst

PEDOT:PSS (Clevios PH1000, Heraeus) filtered using cellulose acetate syringe filters (pore size: 0.45 µm, Advantec MFS, Inc.) was deposited by spin‐coating on pre‐cleaned substrates, followed by annealing at 120°C for 15 min under atmospheric conditions. For the post‐treatments, the PEDOT:PSS films were immersed in concentrated sulfuric acid (> 95%, Duksan Pure Chemicals) for 15 min, thoroughly rinsed with deionized water, and dried at 120 °C for 15 min. To fabricate the crystallized PEDOT:PSS–Pt nanocomposite catalyst, a three‐electrode electrochemical setup was used with a Pt sheet as the counter electrode, saturated Ag/AgCl as the reference electrode, and crystallized PEDOT:PSS film as the working electrode. The plating solution contained 10 mm H_2_PtCl_6_ ∙ 6H_2_O and 10 mm KCl. Pt deposition was performed on crystallized PEDOT:PSS using the pulse‐current electrodeposition technique (applying current: −0.03A cm^−2^, on/off time: 0.3 s/0.1 s, number of the cycle: 12 cycles). For comparison, Pt was deposited on ITO glass substrates using the same method described above but without the crystallized PEDOT:PSS film; this was employed as the control sample.

### 4.2 Film Characterizations

The crystallized PEDOT:PSS and PEDOT:PSS–Pt NPs composites for the electrical conductivity measurement were prepared on a quartz substrate after cleaning by ultrasonication, and the electrical conductivities of the films were measured using a four‐point probe method using a Keithley 2400 source‐meter. The UV–vis absorption spectra were measured using a UV/Vis/NIR spectrophotometer (Agilent Inc., Cary 5000). SEM was performed using a field‐emission scanning electron microscope (Hitachi S‐4700). BSE‐SEM measurement was conducted using an ultra‐high‐resolution field‐emission scanning electron microscope (Thermo Fisher Scientific, Verios 5 UC). The particle size and density were determined using the ImageJ software. The XPS spectra and depth profiles were obtained using a K‐alpha spectrometer (Thermo Fisher Scientific Inc.) with Al Kα radiation. The C 1s peak (284.6 eV) was used as an internal standard to correct for the shift in the binding energy caused by sample charging. HR‐TEM, HAADF‐STEM, and STEM‐EDS images were recorded at 300 kV (Tecnai G^2^ F30 S‐Twin). TEM specimens were prepared on a silicon dioxide system with window grids (nine windows, TEM windows). For the mass loading of Pt, one square centimeter area of the crystallized PEDOT:PSS–Pt NPs composite film on a glass substrate and Pt on an ITO glass substrate were prepared. The samples were analyzed using an inductively coupled plasma optical emission spectrometer (ICP‐OES, Perkin‐Elmer Inc., OPTIMA 7300 DV). For the swelling test, the film thickness was measured using a Park Systems XE‐Bio AFM instrument. To evaluate the change in the thickness of each sample after water immersion, the same position was scanned under dry conditions and re‐scanned in the liquid cell after immersing in water for 20 min.

### 4.3 Electrochemical Characterizations

The electrochemical performance was tested using a three‐electrode system with a PGSTAT304N instrument (Metrohm Autolab). A saturated Ag/AgCl electrode and graphite rod were used as the counter and reference electrodes, respectively. For the saturated Ag/AgCl electrode, the potential against the reversible hydrogen electrode (RHE) was calculated according to the following equation:

$$E\left(vs.RHE\right)=E\left(vs.Ag/AgCl\right)+E_{0}\left(Ag/AgCl\right)+0.0591\times pH$$

where *E*(vs. RHE) is the potential versus RHE, *E*(vs. Ag/AgCl) is the potential versus Ag/AgCl, and *E*_0_(Ag/AgCl) is the potential of Ag/AgCl with respect to the standard hydrogen electrode. As the electrolyte, we employed 0.5 M H_2_SO_4_ (Sigma Aldrich), which was degassed by bubbling with N_2_ to prevent oxygen attack. The cyclic voltammetry (CV) curves were recorded at 50 mV s^−1^. The electrochemically active surface area (ECSA) of the catalysts was determined by the charge involved in the underpotential deposition (UPD) of hydrogen, with a value of 210 µC cm^−2^ for monolayer hydrogen desorption from the Pt surface. Linear sweep voltammetry (LSV) for the HER was performed in N_2_‐saturated 0.5 M H_2_SO_4_ at a scan rate of 10 mV s^−1^ to obtain the polarization curve. The polarization curves were corrected for solution resistance, which was measured by electrochemical impedance spectroscopy (EIS). EIS was performed in 0.5 M H_2_SO_4_ at −0.03 V vs RHE with a frequency ranging from 0.1 Hz to 10 kHz. The turnover frequency (TOF) for HER was calculated according to the following equation:

TOF=j/(F×n×m)

where *j*, *F*, *n*, and m represent current density, the Faraday constant, the number of electrons transferred to generate one molecule of the product (the value of *n* is 2 for HER), and the number of moles of active sites available for the catalysis, respectively. Chronopotentiometry measurements for the HER were carried out at constant current densities of 10 and 20 mA cm^−2^ in 0.5 M H_2_SO_4_. CV for the methanol oxidation was performed in 0.5 M H_2_SO_4_ and 0.1 M CH_3_OH at a scan rate of 50 mV s^−1^. The illumination experiments were performed under an illumination of 1 sun (100 mW cm^−2^) using a 300 W Xe lamp (Newport) with an AM 1.5G filter. Chronoamperometry measurements for the MOR were performed in 0.5 M H_2_SO_4_ containing 1 M CH_3_OH at an applied potential of 0.9 V versus RHE.

## Conflicts of Interest

The authors declare no conflicts of interest.

## Supporting information




**Supporting File**: smll73164‐sup‐0001‐SuppMat.docx.

## Data Availability

The data that support the findings of this study are available from the corresponding author upon reasonable request.

## References

[1] S. Chu and A. Majumdar , “Opportunities and Challenges for a Sustainable Energy Future,” Nature 488 (2012): 294–303, 10.1038/nature11475. 22895334

[2] L. Rößner and M. Armbrüster , “Electrochemical Energy Conversion on Intermetallic Compounds: A Review,” ACS Catalysis 9 (2019): 2018–2062.

[3] A. S. Aricò , P. Bruce , B. Scrosati , J.‐M. Tarascon , and W. van Schalkwijk , “Nanostructured Materials for Advanced Energy Conversion and Storage Devices,” Nature Materials 4 (2005): 366–377. 15867920 10.1038/nmat1368

[4] X. Zhang , X. Cheng , and Q. Zhang , “Nanostructured Energy Materials for Electrochemical Energy Conversion and Storage: A Review,” Journal of Energy Chemistry 25 (2016): 967–984, 10.1016/j.jechem.2016.11.003.

[5] Z. W. Seh , J. Kibsgaard , C. F. Dickens , I. Chorkendorff , J. K. Nørskov , and T. F. Jaramillo , “Combining Theory and Experiment in Electrocatalysis: Insights Into Materials Design,” Science 355 (2017): aad4998, 10.1126/science.aad4998. 28082532

[6] W. Lv , Z. Li , Y. Deng , Q.‐H. Yang , and F. Kang , “Graphene‐Based Materials for Electrochemical Energy Storage Devices: Opportunities and Challenges,” Energy Storage Materials 2 (2016): 107–138, 10.1016/j.ensm.2015.10.002.

[7] G. Zhao , K. Rui , S. X. Dou , and W. Sun , “Heterostructures for Electrochemical Hydrogen Evolution Reaction: A Review,” Advanced Functional Materials 28 (2018): 1803291, 10.1002/adfm.201803291.

[8] M. Tahir , L. Pan , F. Idrees , et al., “Electrocatalytic Oxygen Evolution Reaction for Energy Conversion and Storage: A Comprehensive Review,” Nano Energy 37 (2017): 136–157, 10.1016/j.nanoen.2017.05.022.

[9] X. Wang , Z. Li , Y. Qu , et al., “Review of Metal Catalysts for Oxygen Reduction Reaction: From Nanoscale Engineering to Atomic Design,” Chemistry 5 (2019): 1486–1511, 10.1016/j.chempr.2019.03.002.

[10] J. Qiao , Y. Liu , F. Hong , and J. Zhang , “A Review of Catalysts for the Electroreduction of Carbon Dioxide to Produce Low‐Carbon Fuels,” Chemical Society Reviews 43 (2014): 631–675, 10.1039/C3CS60323G. 24186433

[11] X. L. Tian , L. Wang , P. Deng , Y. Chen , and B. Y. Xia , “Research Advances in Unsupported Pt‐Based Catalysts for Electrochemical Methanol Oxidation,” Journal of Energy Chemistry 26 (2017): 1067–1076, 10.1016/j.jechem.2017.10.009.

[12] J. Bai , D. Liu , J. Yang , and Y. Chen , “Nanocatalysts for Electrocatalytic Oxidation of Ethanol,” Chemsuschem 12 (2019): 2117–2132, 10.1002/cssc.201803063. 30834720

[13] Z. Peng and H. Yang , “Designer Platinum Nanoparticles: Control of Shape, Composition in Alloy, Nanostructure and Electrocatalytic Property,” Nano Today 4 (2009): 143–164, 10.1016/j.nantod.2008.10.010.

[14] X. Ren , Q. Lv , L. Liu , et al., “Current Progress of Pt and Pt‐Based Electrocatalysts Used for Fuel Cells,” Sustainable Energy & Fuels 4 (2020): 15–30, 10.1039/C9SE00460B.

[15] D. Liu , L. Li , and T. You , “Superior Catalytic Performances of Platinum Nanoparticles Loaded Nitrogen‐Doped Graphene Toward Methanol Oxidation and Hydrogen Evolution Reaction,” Journal of Colloid and Interface Science 487 (2017): 330–335, 10.1016/j.jcis.2016.10.038. 27792940

[16] Q. Liu , L. Du , G. Fu , et al., “Structurally Ordered Fe _3_ Pt Nanoparticles on Robust Nitride Support as a High Performance Catalyst for the Oxygen Reduction Reaction,” Advanced Energy Materials 9 (2019): 1803040, 10.1002/aenm.201803040.

[17] Y. Jiao , Y. Zheng , M. Jaroniec , and S. Z. Qiao , “Design of Electrocatalysts for Oxygen‐ and Hydrogen‐Involving Energy Conversion Reactions,” Chemical Society Reviews 44 (2015): 2060–2086, 10.1039/C4CS00470A. 25672249

[18] Y. Nie , L. Li , and Z. Wei , “Recent Advancements in Pt and Pt‐Free Catalysts for Oxygen Reduction Reaction,” Chemical Society Reviews 44 (2015): 2168–2201, 10.1039/C4CS00484A. 25652755

[19] M. D. Bhatt and J. Y. Lee , “Advancement of Platinum (Pt)‐Free (Non‐Pt Precious Metals) and/or Metal‐Free (Non‐Precious‐Metals) Electrocatalysts in Energy Applications: a Review and Perspectives,” Energy & Fuels 34 (2020): 6634–6695, 10.1021/acs.energyfuels.0c00953.

[20] N. Cheng , S. Stambula , D. Wang , et al., “Platinum Single‐Atom and Cluster Catalysis of the Hydrogen Evolution Reaction,” Nature Communications 7 (2016): 13638, 10.1038/ncomms13638. PMC5141386 27901129

[21] L. Liu , D. M. Meira , R. Arenal , P. Concepcion , A. V. Puga , and A. Corma , “Determination of the Evolution of Heterogeneous Single Metal Atoms and Nanoclusters Under Reaction Conditions: Which Are the Working Catalytic Sites?,” ACS Catalysis 9 (2019): 10626–10639, 10.1021/acscatal.9b04214. 31840008 PMC6902617

[22] L. Zhang , K. Doyle‐Davis , and X. Sun , “Pt‐Based Electrocatalysts With High Atom Utilization Efficiency: From Nanostructures to Single Atoms,” Energy & Environmental Science 12 (2019): 492–517, 10.1039/C8EE02939C.

[23] L. Liu and A. Corma , “Metal Catalysts for Heterogeneous Catalysis: From Single Atoms to Nanoclusters and Nanoparticles,” Chemical Reviews 118 (2018): 4981–5079, 10.1021/acs.chemrev.7b00776. 29658707 PMC6061779

[24] H. Ou , D. Wang , and Y. Li , “How to Select Effective Electrocatalysts: Nano or Single Atom?,” Nano Select 2 (2021): 492–511, 10.1002/nano.202000239.

[25] G.‐F. Wei and Z.‐P. Liu , “Restructuring and Hydrogen Evolution on Pt Nanoparticle,” Chemical Science 6 (2015): 1485–1490, 10.1039/C4SC02806F. 29560237 PMC5811100

[26] K. Kodama , T. Nagai , A. Kuwaki , R. Jinnouchi , and Y. Morimoto , “Challenges in Applying Highly Active Pt‐Based Nanostructured Catalysts for Oxygen Reduction Reactions to Fuel Cell Vehicles,” Nature Nanotechnology 16 (2021): 140–147, 10.1038/s41565-020-00824-w. 33479539

[27] S. Yoo , Y. Kim , Y. Yoon , M. Karuppannan , O. J. Kwon , and T. Lim , “Encapsulation of Pt Nanocatalyst With N‐Containing Carbon Layer for Improving Catalytic Activity and Stability in the Hydrogen Evolution Reaction,” International Journal of Hydrogen Energy 46 (2021): 21454–21461, 10.1016/j.ijhydene.2021.03.225.

[28] D. V. Esposito , S. T. Hunt , A. L. Stottlemyer , et al., “Low‐Cost Hydrogen‐Evolution Catalysts Based on Monolayer Platinum on Tungsten Monocarbide Substrates,” Angewandte Chemie International Edition 49 (2010): 9859–9862, 10.1002/anie.201004718. 20886586

[29] H. Wei , K. Huang , D. Wang , et al., “Iced Photochemical Reduction to Synthesize Atomically Dispersed Metals by Suppressing Nanocrystal Growth,” Nature Communications 8 (2017): 1490, 10.1038/s41467-017-01521-4. PMC5684195 29133795

[30] Z. Wang , J. Yang , J. Gan , et al., “Electrochemical Conversion of Bulk Platinum Into Platinum Single‐Atom Sites for the Hydrogen Evolution Reaction,” Journal of Materials Chemistry A 8 (2020): 10755–10760, 10.1039/D0TA02351E.

[31] C. Xia , Y. Qiu , Y. Xia , et al., “General Synthesis of Single‐Atom Catalysts With High Metal Loading Using Graphene Quantum Dots,” Nature Chemistry 13 (2021): 887–894, 10.1038/s41557-021-00734-x. 34168326

[32] A. J. M. Mackus , M. A. Verheijen , N. Leick , A. A. Bol , and W. M. M. Kessels , “Influence of Oxygen Exposure on the Nucleation of Platinum Atomic Layer Deposition: Consequences for Film Growth, Nanopatterning, and Nanoparticle Synthesis,” Chemistry of Materials 25 (2013): 1905–1911, 10.1021/cm400562u.

[33] X.‐F. Yang , A. Wang , B. Qiao , J. Li , J. Liu , and T. Zhang , “Single‐Atom Catalysts: A New Frontier in Heterogeneous Catalysis,” account Chemical Research 46 (2013): 1740–1748. 10.1021/ar300361m 23815772

[34] H. Li , C. Chen , D. Yan , et al., “Interfacial Effects in Supported Catalysts for Electrocatalysis,” Journal of Materials Chemistry A 7 (2019): 23432–23450, 10.1039/C9TA04888J.

[35] T. W. van Deelen , C. Hernández Mejía , and K. P. de Jong , “Control of Metal‐Support Interactions in Heterogeneous Catalysts to Enhance Activity and Selectivity,” Nature Catalysis 2 (2019): 955–970, 10.1038/s41929-019-0364-x.

[36] J. Gan , Z. Huang , W. Luo , et al., “Platelet Carbon Nanofibers as Support of Pt‐CoO Electrocatalyst for Superior Hydrogen Evolution,” Journal of Energy Chemistry 52 (2021): 33–40, 10.1016/j.jechem.2020.04.036.

[37] W.‐J. Liu , X. Hu , H.‐C. Li , and H.‐Q. Yu , “Pseudocapacitive Ni‐Co‐Fe Hydroxides/N‐Doped Carbon Nanoplates‐Based Electrocatalyst for Efficient Oxygen Evolution,” Small 14 (2018): 1801878, 10.1002/smll.201801878. 30063288

[38] C.‐T. Shen , K.‐W. Wang , C.‐J. Tseng , K.‐R. Lee , and Y.‐J. Hsueh , “The Oxygen Reduction Reaction of Ordered Porous Carbon‐Supported PtSn Catalysts,” RSC Advances 6 (2016): 44205–44211.

[39] A. H. Ghanim , J. G. Koonce , B. Hasa , et al., “Low‐Loading of Pt Nanoparticles on 3D Carbon Foam Support for Highly Active and Stable Hydrogen Production,” Frontiers in Chemistry 6 (2018): 523, 10.3389/fchem.2018.00523. 30460227 PMC6232265

[40] J. Choi , J. Kim , P. Wagner , et al., “Energy Efficient Electrochemical Reduction of CO 2 to CO Using a Three‐Dimensional Porphyrin/Graphene Hydrogel,” Energy & Environmental Science 12 (2019): 747–755, 10.1039/C8EE03403F.

[41] X. Wang , J. Deng , Y. Liu , et al., “Mesoporous Si‐WO3‐Supported Pt Catalysts With High Catalytic Performance and Excellent Water Resistance for Toluene Oxidation,” Catalysis Today 432 (2024): 114650, 10.1016/j.cattod.2024.114650.

[42] S.‐M. Kim , C.‐H. Kim , Y. Kim , et al., “Influence of PEDOT:PSS Crystallinity and Composition on Electrochemical Transistor Performance and Long‐Term Stability,” Nature Communications 9 (2018): 3858, 10.1038/s41467-018-06084-6. PMC6155079 30242224

[43] S.‐S. Kim , Y.‐C. Nah , Y.‐Y. Noh , J. Jo , and D.‐Y. Kim , “Electrodeposited Pt for Cost‐Efficient and Flexible Dye‐Sensitized Solar Cells,” Electrochimica Acta 51 (2006): 3814–3819, 10.1016/j.electacta.2005.10.047.

[44] P. Wang , L. Zong , Z. Guan , Q. Li , and J. Yang , “PtNi Alloy Cocatalyst Modification of Eosin Y‐Sensitized G‐C_3_N_4_/GO Hybrid for Efficient Visible‐Light Photocatalytic Hydrogen Evolution,” Nanoscale Research Letters 13 (2018): 33, 10.1186/s11671-018-2448-y. 29396656 PMC5796927

[45] C. Cui , R. Cheng , H. Zhang , et al., “Ultrastable MXene@Pt/SWCNTs' Nanocatalysts for Hydrogen Evolution Reaction,” Advanced Functional Materials 30 (2020): 2000693, 10.1002/adfm.202000693.

[46] N. Kim , S. Kee , S. H. Lee , et al., “Highly Conductive PEDOT:PSS Nanofibrils Induced by Solution‐Processed Crystallization,” Advanced Materials 26 (2014): 2268–2272, 10.1002/adma.201304611. 24338693

[47] D. Pantea , H. Darmstadt , S. Kaliaguine , and C. Roy , “Electrical Conductivity of Conductive Carbon Blacks: Influence of Surface Chemistry and Topology,” Applied Surface Science 217 (2003): 181–193, 10.1016/S0169-4332(03)00550-6.

[48] C.‐P. Lo and V. Ramani , “SiO _2_ –RuO _2_: A Stable Electrocatalyst Support,” ACS Applied Materials & Interfaces 4 (2012): 6109–6116, 10.1021/am3017416. 23057537

[49] E. Kang , S. An , S. Yoon , J. K. Kim , and J. Lee , “Ordered Mesoporous WO_3−X_ Possessing Electronically Conductive Framework Comparable to Carbon Framework Toward Long‐Term Stable Cathode Supports for Fuel Cells,” Journal of Materials Chemistry 20 (2010): 7416, 10.1039/c0jm00227e.

[50] J.‐J. Shao , Z.‐J. Li , C. Zhang , L.‐F. Zhang , and Q.‐H. Yang , “A Wavy Graphene/Platinum Hybrid With Increased Electroactivity for the Methanol Oxidation Reaction,” Journal of Materials Chemistry A 2 (2014): 1940–1946, 10.1039/C3TA14134A.

[51] Y. Li , T. Bian , J. Du , et al., “Facile Synthesis of High‐Quality Pt Nanostructures With a Controlled Aspect Ratio for Methanol Electro‐Oxidation,” CrystEngComm 16 (2014): 8340–8343, 10.1039/C4CE00713A.

[52] J. Bi , P. Gao , B. Wang , et al., “Intrinsic Insight on Localized Surface Plasmon Resonance Enhanced Methanol Electro‐Oxidation Over a Au@AgPt Hollow Urchin‐Like Nanostructure,” Journal of Materials Chemistry A 8 (2020): 6638–6646, 10.1039/C9TA13567G.

[53] Y. Kim , A. Lund , H. Noh , et al., “Robust PEDOT:PSS Wet‐Spun Fibers for Thermoelectric Textiles,” Macromolecular Materials and Engineering 305 (2020): 1900749, 10.1002/mame.201900749.

