# Supplementary material for: A Highly Porous Nanofibrillar PEDOT:PSS Matrix for Beyond‐Surface Precious‐Metal Utilization and Volumetric Electrocatalysis
Source: Small. 2026 Mar 31;22(27):e14951. doi: 10.1002/smll.202514951 (PMC13173316; doi:10.1002/smll.202514951)
Supplement: Supplementary file 1 — Supporting File: smll73164‐sup‐0001‐SuppMat.docx. [file SMLL-22-e14951-s001.docx]

**Supporting Information**

**A Highly Porous Nanofibrillar PEDOT:PSS Matrix for Beyond-Surface Precious-Metal Utilization and Volumetric Electrocatalysis**

Da-Young Lee^1,2^, Hye-Min Shin^1^, Ji Hwan Kim^1^, and Myung-Han Yoon^1,2*^

^1^*Department of Materials Science and Engineering, Gwangju Institute of Science and Technology (GIST), 123 Cheomdangwagi-ro, Buk-gu, Gwangju 61005, Republic of Korea*

^2^*GIST InnoCORE AI-Nano Convergence Institute for Early Detection of Neurodegenerative Diseases, Gwangju Institute of Science and Technology (GIST), Gwangju 61005, Republic of Korea*

*Corresponding author: Prof. Myung-Han Yoon (mhyoon@gist.ac.kr)


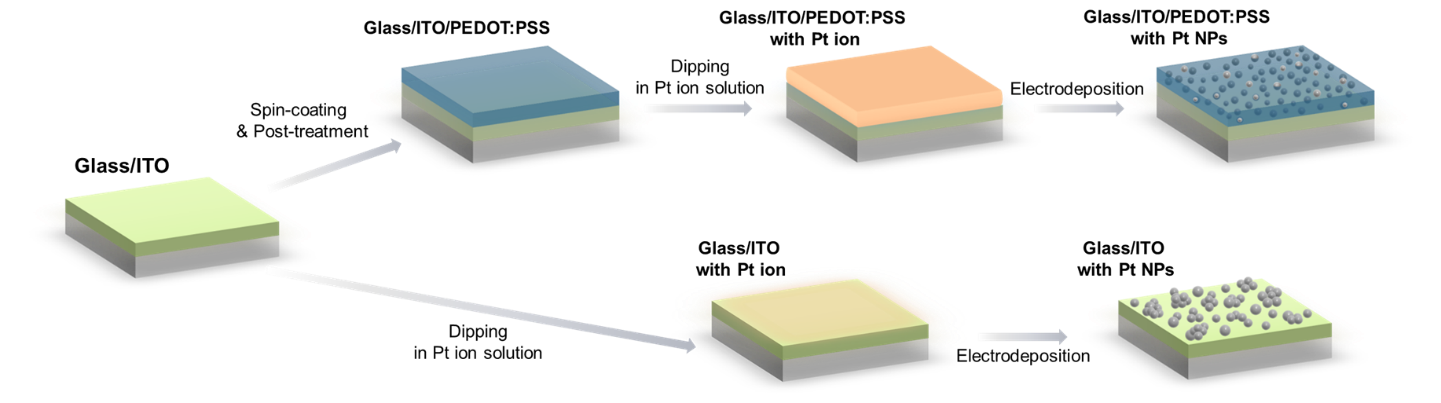


Figure S1. Schematic illustration of the fabrication processes for crystallized PEDOT:PSS–Pt NPs composites and Pt NPs deposited without crystallized PEDOT:PSS.


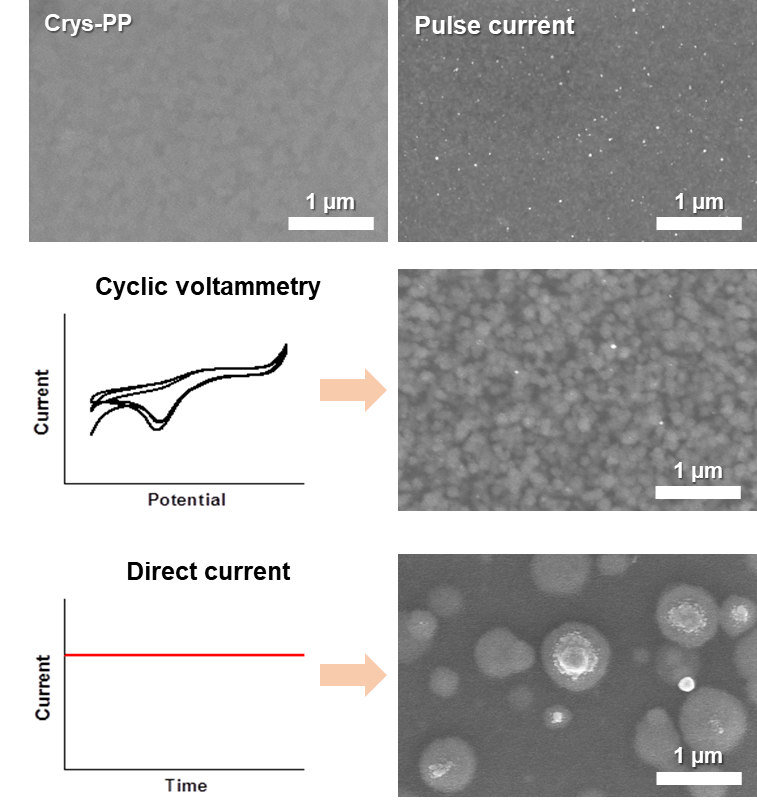


Figure S2. (a) SEM images of Pt nanoparticles prepared using different electrodeposition methods.


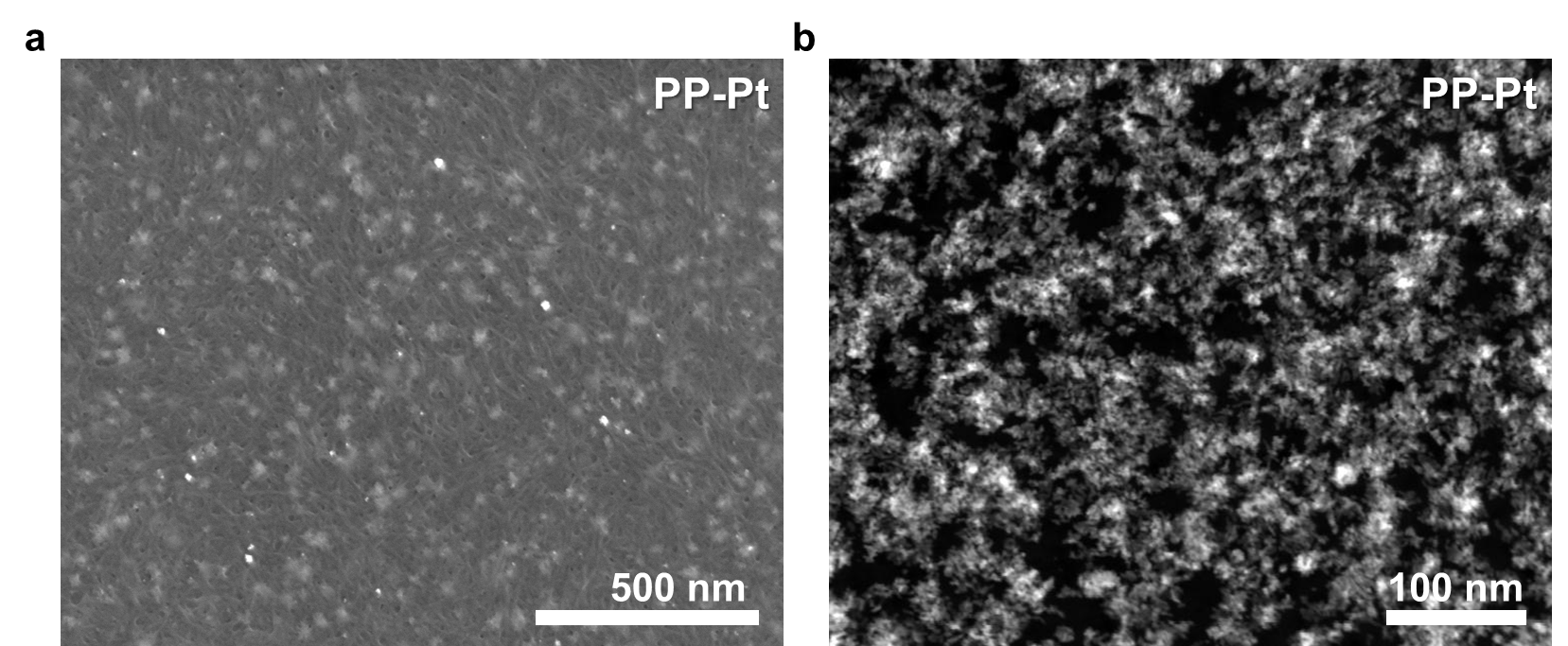


Figure S3. (a) BSE-SEM image and (b) HADDF-STEM image of PP–Pt.


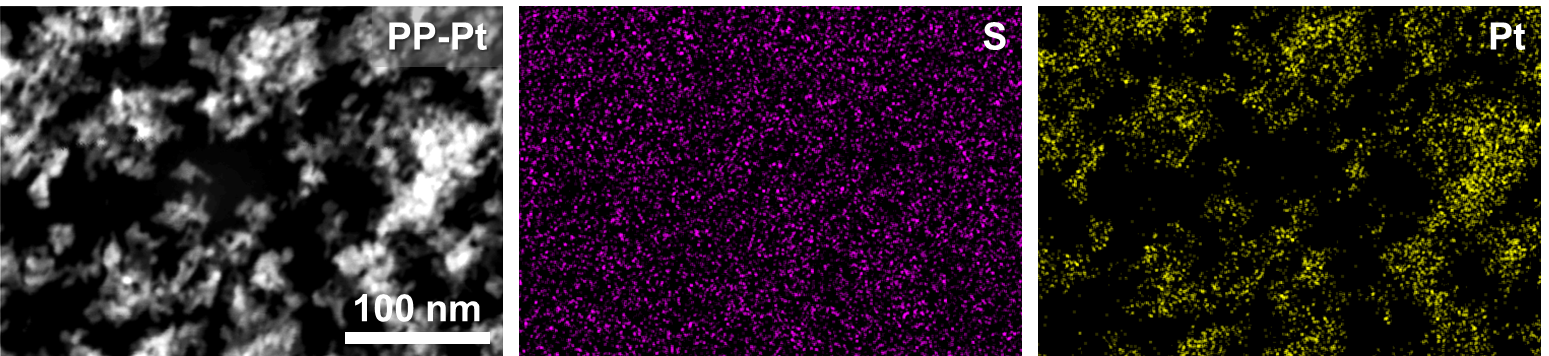


Figure S4. HAADF-STEM image and corresponding STEM-EDS elemental maps of S and Pt of the PP–Pt.


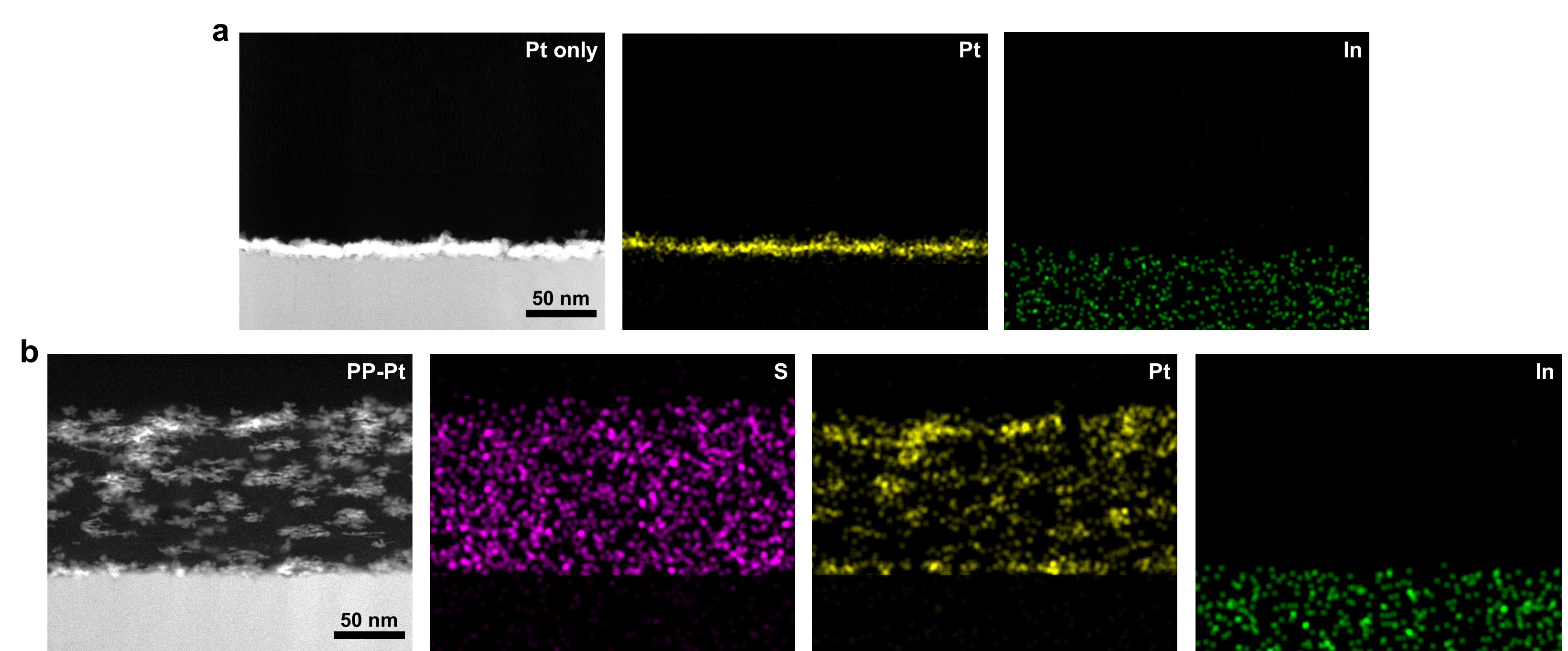


Figure S5. Cross-sectional HAADF-STEM image and corresponding STEM-EDS elemental maps of (a) Pt only and (b) PP–Pt.


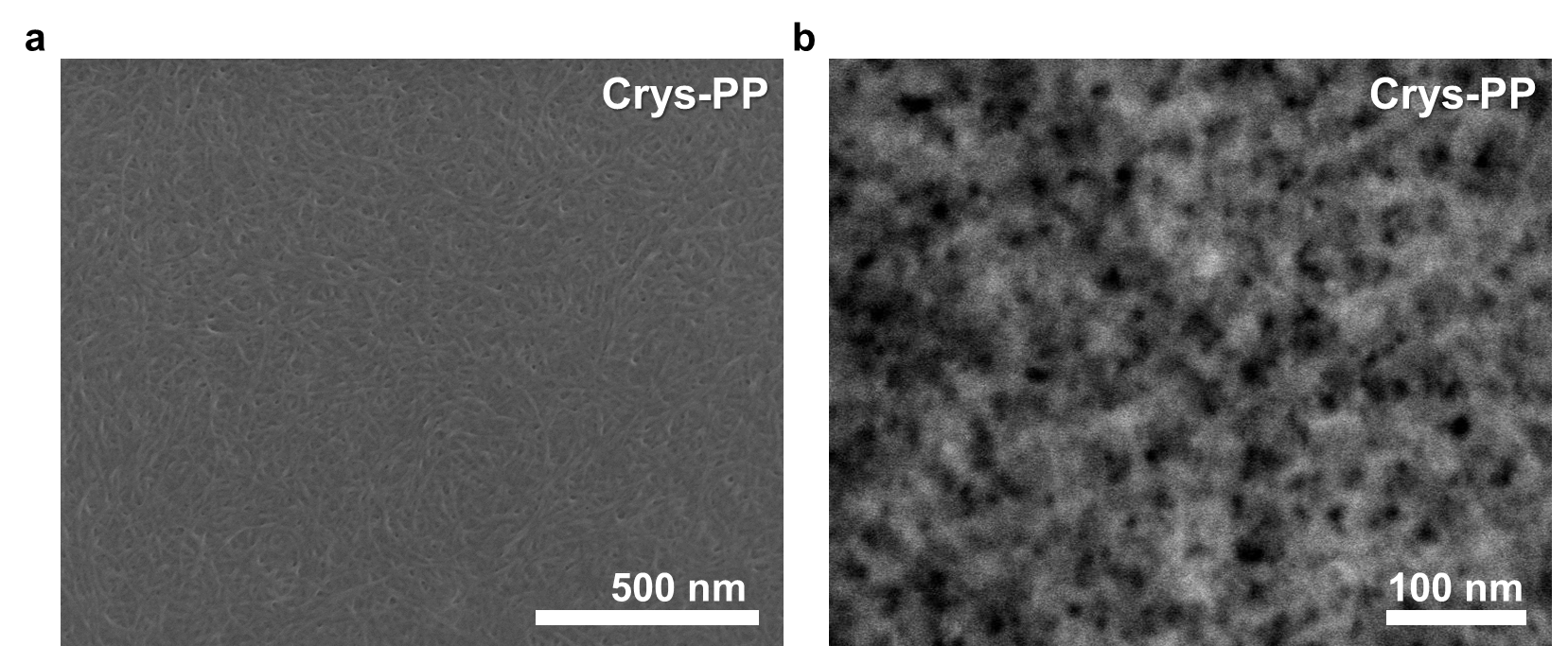


Figure S6. (a) BSE-SEM image and (b) HADDF-STEM image of crystallized PEDOT:PSS film only (without Pt electrodeposition).


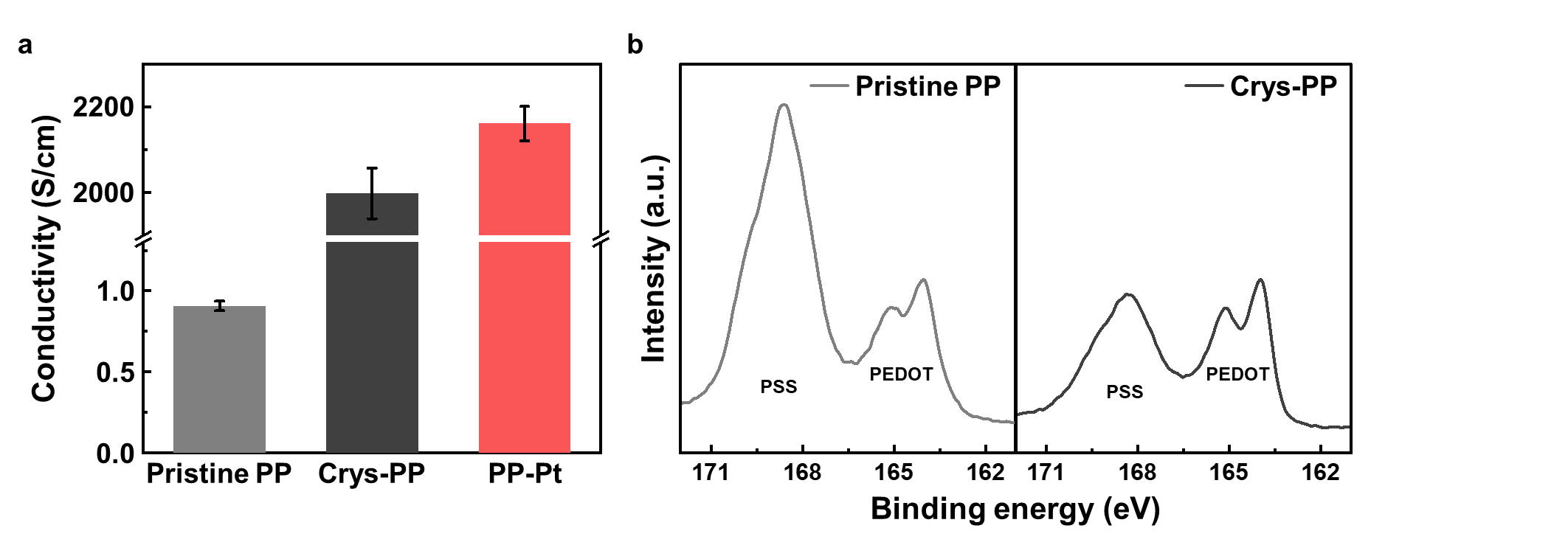


Figure S7. (a) Plots of electrical conductivity of pristine PEDOT:PSS, crystallized PEDOT:PSS (Crys-PP), and PP-Pt. (b) XPS S 2*p* peaks of pristine PEDOT:PSS and crystallized PEDOT:PSS.


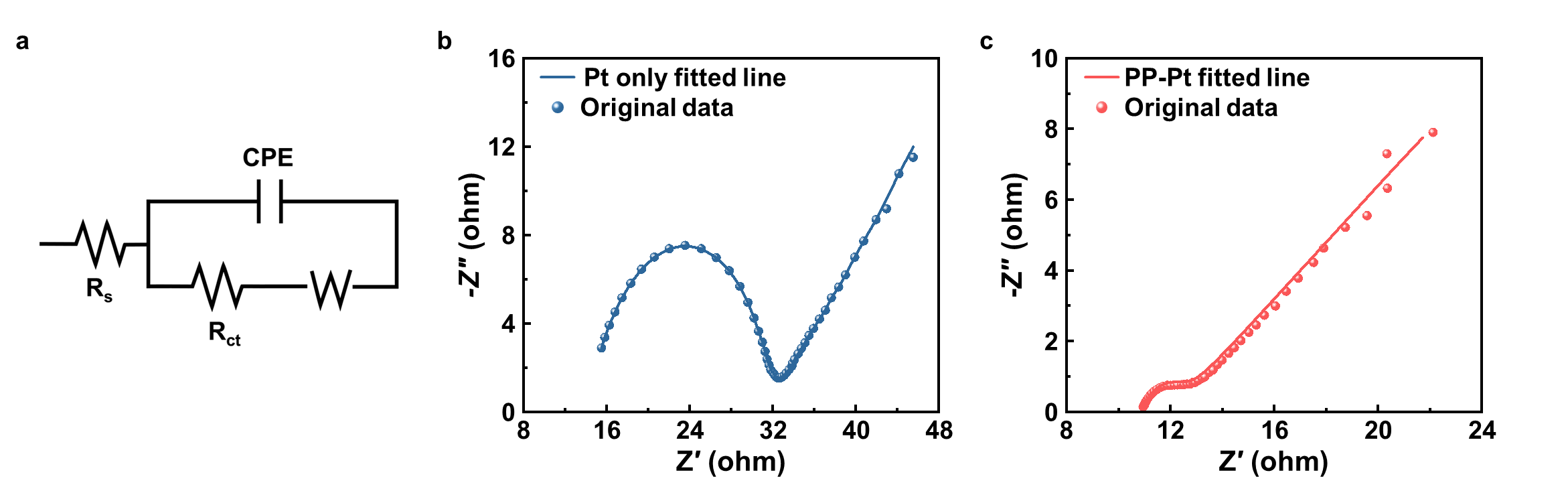


Figure S8. (a) Equivalent circuit model employed for fitting the impedance spectra. (b) Nyquist plot of Pt. (c) Nyquist plot of PP–Pt.


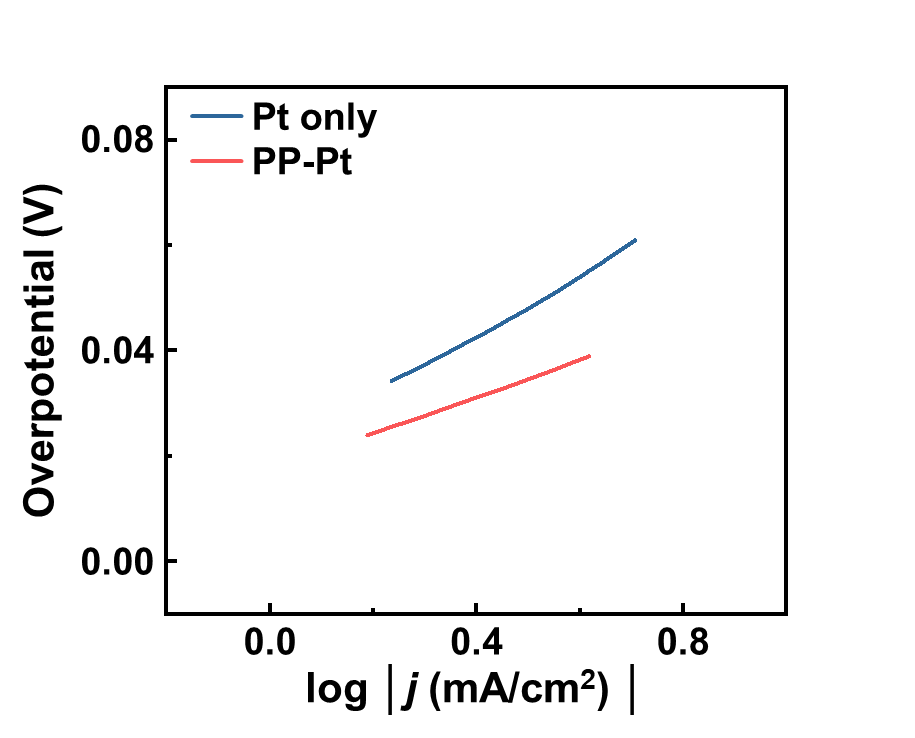


Figure S9. Tafel plots of the hydrogen evolution reaction for Pt only and PP–Pt electrodes.


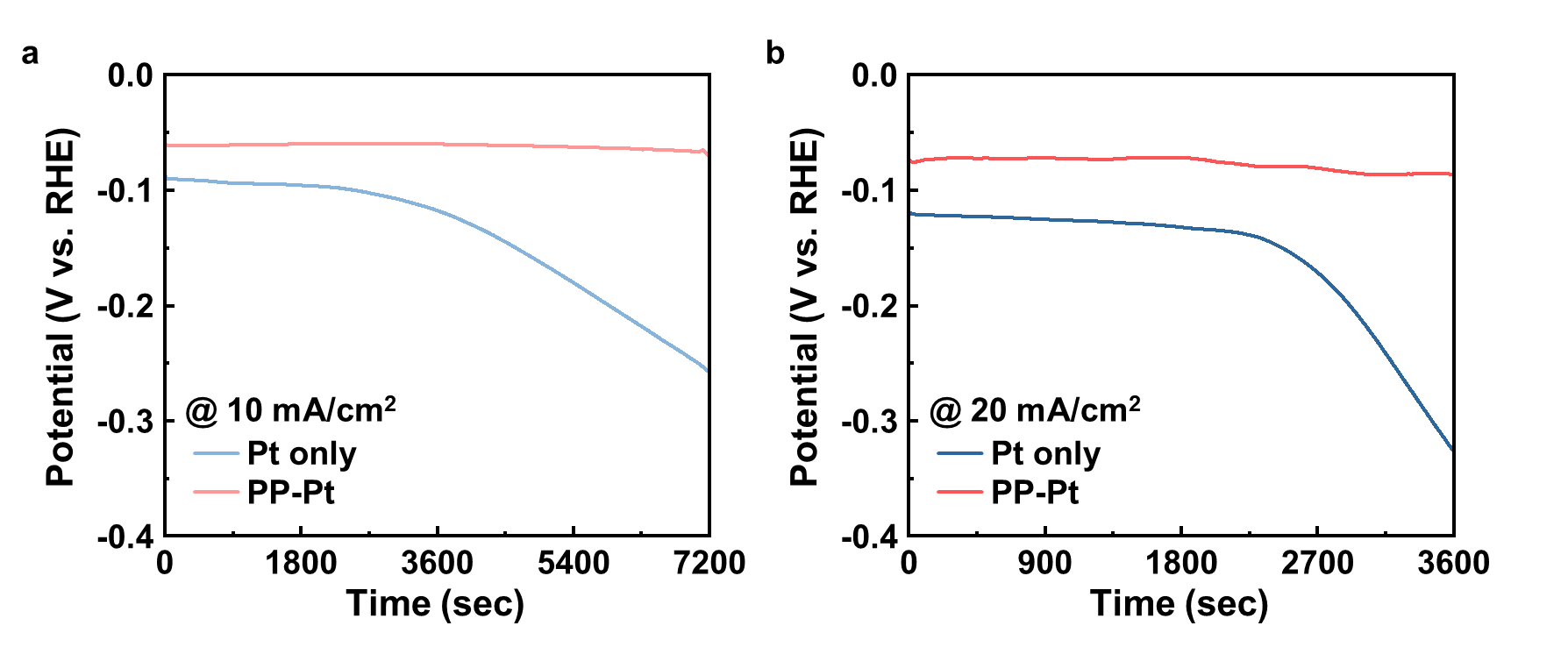


Figure S10. Chronopotentiometry measurements of Pt only (blue) and PP-Pt (red) electrodes at (a) 10 mA cm^-2^ and (b) 20 mA cm^-2^ (0.5 M H_2_SO_4_, N_2_ purged).


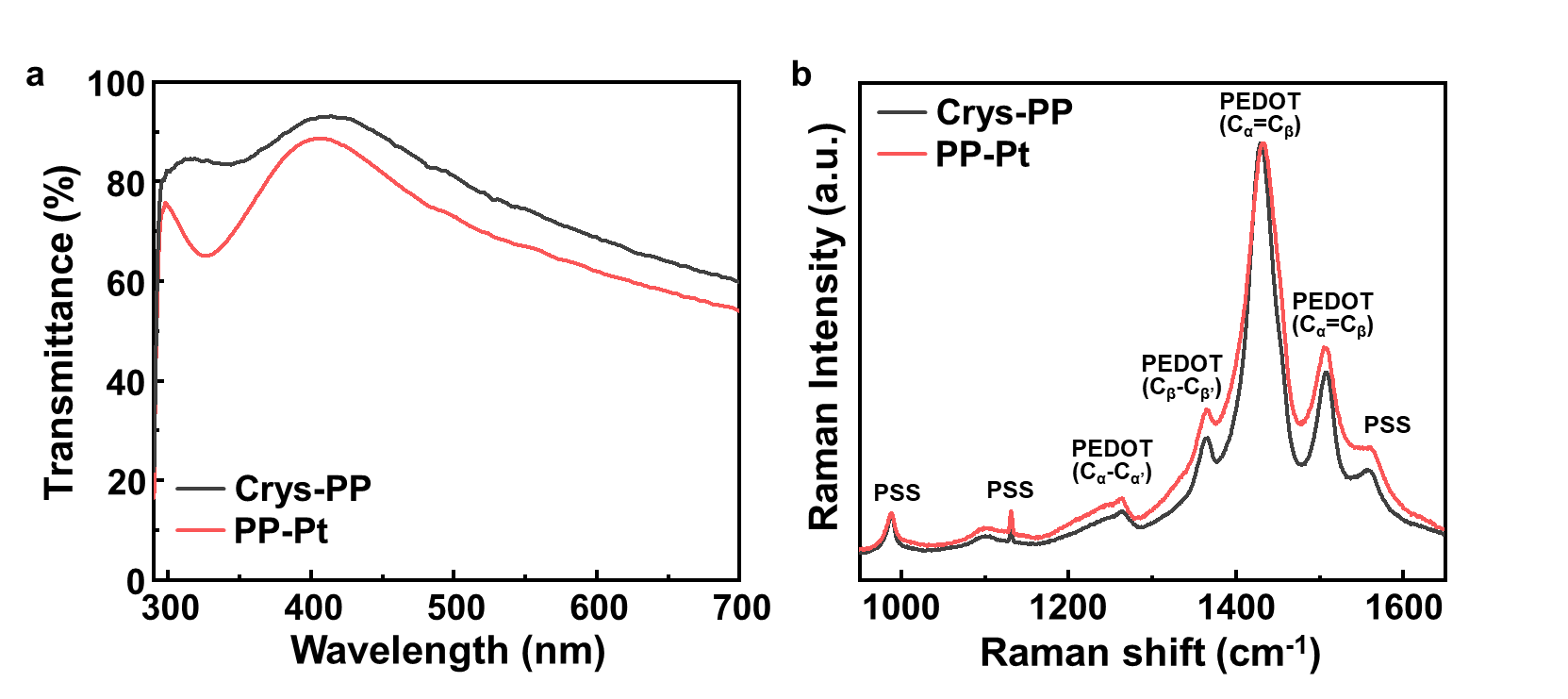


Figure S11. (a) UV-Vis transmittance spectra and (b) Raman spectra of crystallized PEDOT:PSS (Crys-PP) and PP-Pt.


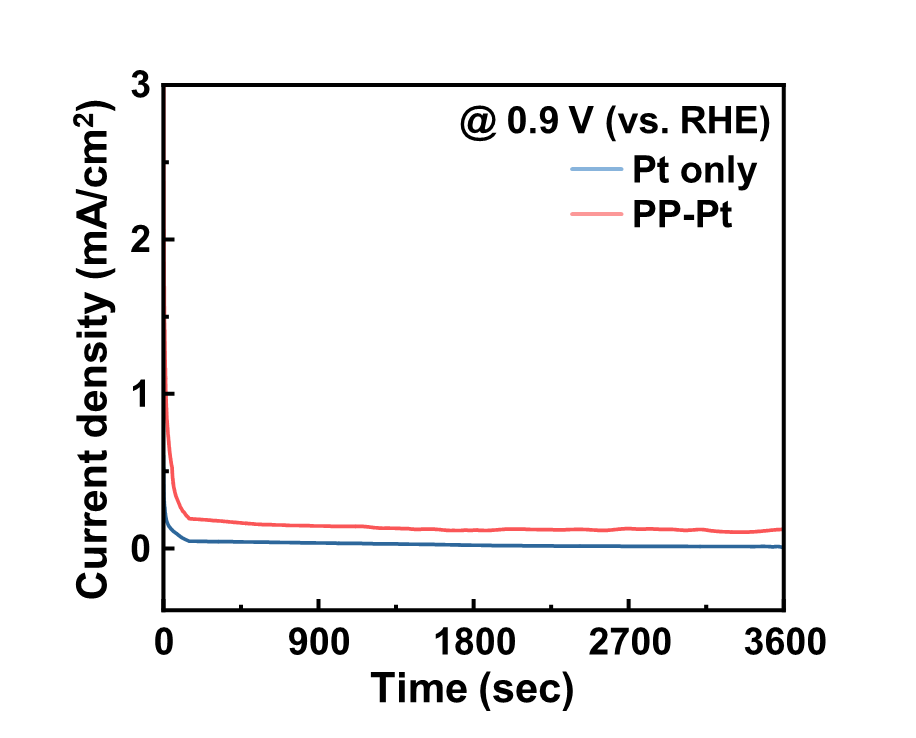


Figure S12. Chronoamperometry measurements of Pt only (blue) and PP-Pt (red) electrodes at 0.9 V vs. RHE (0.5 M H_2_SO_4_, N_2_ purged)


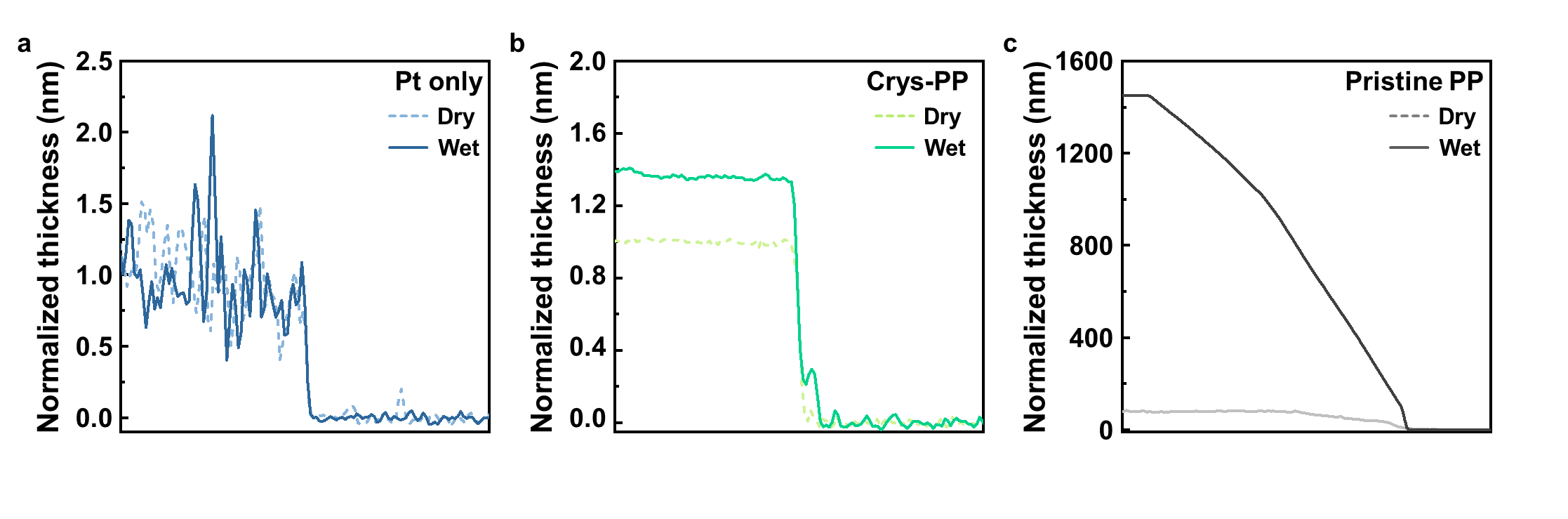


Figure S13. Comparison of dry and hydrated film thicknesses for (a) Pt, (b) crystallized PEDOT:PSS film (Crys-PP), and (c) pristine PEDOT:PSS film.


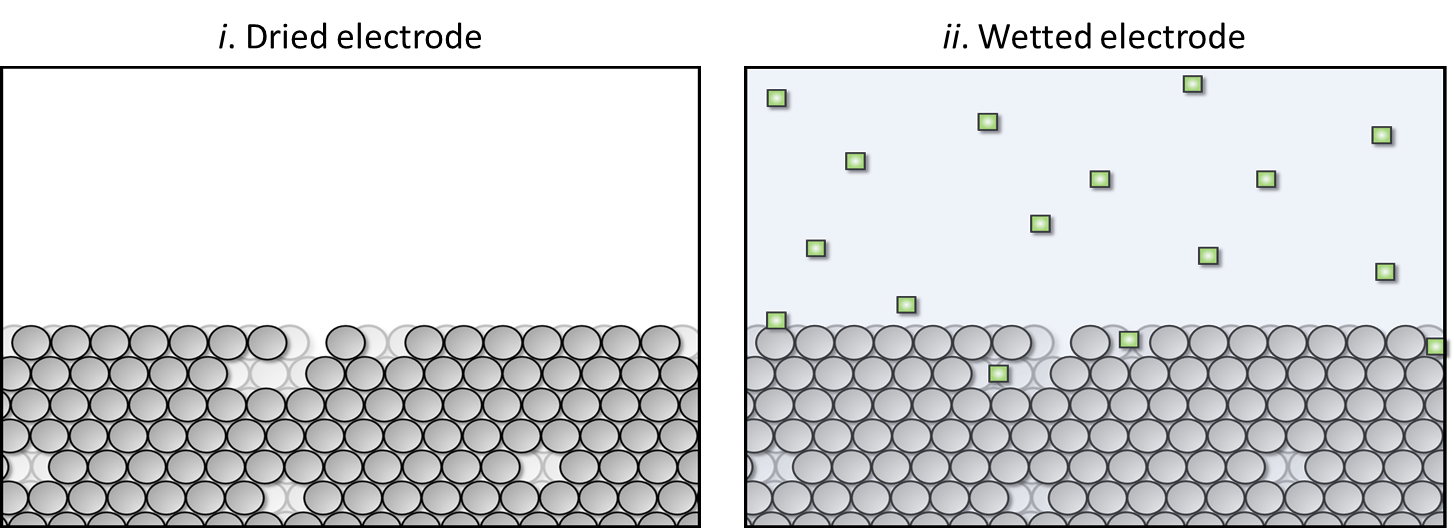


Figure S14. Schematic illustration of the catalytic reaction pathway for Pt.


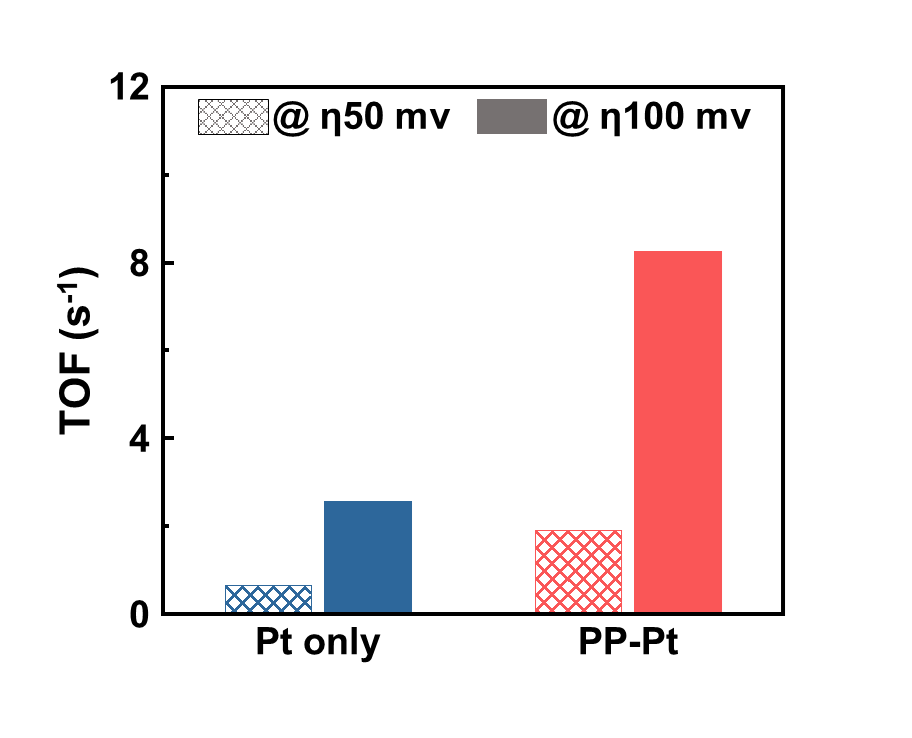


Figure S15. TOF of the hydrogen evolution reaction for Pt only and PP-Pt electrodes.

| **Catalyst** | **TOF**  **(S^-1^)** | **Overpotential**  **(mV)** | **Reference** |
| --- | --- | --- | --- |
| PT/NPC | 3.67 | 80 | *Carbon*  **2022**, 196, 621-632 |
| 10Pt@HN-BC | 1.56 | 50 | *Int. J. Hydrogen Energy* **2018**, 43, 6167-6176 |
| PN NCs-MXene/CP | 7.09 | 100 | *J. Electrochem. Soc.*  **2021**, 168, 096504 |
| Pt@PCM | 3.8 | 100 | *Sci. Adv.*  **2018**, 4, eaao6657 |
| Pt1/NMHCS | 4.47 | 100 | *Adv. Mater.*  **2021**, 33, 2008599 |
| **Pt only** | 0.6 | 50 | **This work** |
| **Pt only** | 2.6 | 100 | **This work** |
| **PP-Pt** | 1.9 | 50 | **This work** |
| **PP-Pt** | 8.3 | 100 | **This work** |

Table S1. Comparison of TOF for HER between this work and previously reported Pt-based electrocatalysts.

| **Catalyst** | **TOF**  **(S^-1^)** | **Overpotential**  **(mV)** | **Reference** |
| --- | --- | --- | --- |
| Pt@PCM | 65 | 105 | *Sci. Adv.*  **2018**, 4, eaao6657 |
| Pt1/NMHCS | 56 | 40 | *Adv. Mater.*  **2021**, 33, 2008599 |
| PtNP/NMHCS | 68 | 75 | *Adv. Mater.*  **2021**, 33, 2008599 |
| 3% Pt-MoS2 | 76.2 | 67.4 | *Nano Energy*  **2022**, 94, 106913 |
| Pt-MoS2- 1hr | 41 | 78.5 | *Chem. Commun.*  **2021**, 57, 2879-2882 |
| TBA-Ti3C2Tx-Pt-20 | 65 | 55 | *Chem. Eng.*  ***2019****, 7, 4266-4273* |
| 0.5 wt% Pt Cs/MoO2 NSs-L | 32.6 | 38 | *Nano Energy*  ***2019****, 62, 127-135* |
| 0.5 wt% Pt NPs/MoO2 NSs-L | 68.1 | 127 | *Nano Energy*  ***2019****, 62, 127-135* |
| PtNx/TiO2 | 34 | 67 | *Nano Energy*  ***2020****, 73, 104739* |
| Pt/PANI-Ti3C2Tx | 39 | 52 | *. Electrochem. Soc.*  ***2022****, 169, 036507* |
| Pt-MoS2 | 96 | 60 | *Energy Environ. Sci.*  **2015**, 8, 1594-1601 |
| Pt/LSG200 | 72 | 131 | *J. Mater. Chem. A*  **2017**, 5, 20422–20427 |
| Pt/SnS2-1500C | 69 | 117 | *ACS Appl. Mater. Interfaces*  **2017**, 9, 37750–37759 |
| Pt NP/m-WO3-x | 102 | 152 | *Angew. Chem. Int. Ed.*  **2019**, 58, 16038–16042 |
| Pt/def-WO3@CFC | 61 | 42 | *J. Mater. Chem. A*  **2019**, 7, 6285–6293 |
| **Pt only** | 57 | 84 | **This work** |
| **PP-Pt** | 33 | 54 | **This work** |

Table S2. Comparison of overpotentials and Tafel slopes for HER between this work and previously reported Pt-based electrocatalysts.
